# Mathematical modeling of diurnal patterns of carbon allocation to shoot and root in *Arabidopsis thaliana*

**DOI:** 10.1038/s41540-018-0080-1

**Published:** 2019-01-24

**Authors:** Lisa Küstner, Thomas Nägele, Arnd G. Heyer

**Affiliations:** 1 University of Stuttgart, Institute of Biomaterials and Biomolecular Systems, Department of Plant Biotechnology, Pfaffenwaldring 57, 70569 Stuttgart, Germany; 2 Ludwig-Maximilians-University Munich, Department Biology I, Plant Evolutionary Cell Biology, Großhaderner Str. 2-4, 82152 Planegg-Martinsried, Germany

## Abstract

We developed a mathematical model to simulate dynamics of central carbon metabolism over complete diurnal cycles for leaves of *Arabidopsis thaliana* exposed to either normal (120 µmol m^−2^ s^−1^) or high light intensities (1200 µmol m^−^^2^ s^−1^). The main objective was to obtain a high-resolution time series for metabolite dynamics as well as for shoot structural carbon formation (compounds with long residence time) and assimilate export of aerial organs to the sink tissue. Model development comprised a stepwise increment of complexity to finally approach the *in vivo* situation. The correct allocation of assimilates to either sink export or shoot structural carbon formation was a central goal of model development. Diurnal gain of structural carbon was calculated based on the daily increment in total photosynthetic carbon fixation, and this was the only parameter for structural carbon formation implemented in the model. Simulations of the dynamics of central metabolite pools revealed that shoot structural carbon formation occurred solely during the light phase but not during the night. The model allowed simulation of shoot structural carbon formation as a function of central leaf carbon metabolism under different environmental conditions without structural modifications. Model simulations were performed for the accession Landsberg *erecta* (Ler) and its hexokinase null-mutant *gin2-1*. This mutant displays a slow growth phenotype especially at increasing light intensities. Comparison of simulations revealed that the retarded shoot growth in the mutant resulted from an increased assimilate transport to sink organs. Due to its central function in sucrose cycling and sugar signaling, our findings suggest an important role of hexokinase-1 for carbon allocation to either shoot growth or assimilate export.

## “Mathematical modeling of diurnal patterns of carbon allocation to shoot and root in Arabidopsis thaliana.”

Allocation of carbon fixed in plant photosynthesis to growth under changing environmental conditions is of high interest for future crop production in the context of an ongoing climate change. In this article, Lisa Kuster with her colleagues used the model plant *Arabidopsis thaliana* to simulate carbon distribution between shoot and root under different light conditions. This revealed biomass formation to occur only during daytime, while metabolism is limited to maintenance at night. Plant leaves appear to respire fixed carbon during the entire day, probably to support amino acid synthesis. A defect in the sugar sensor hexokinase-1 (HXK1) caused a strong bias towards carbon allocation to root supply in the *A. thaliana* mutant *gin2-1*., explaining the retarded growth phenotype of this mutant especially under rising light intensities.

## Introduction

With rising interest in plant biomass for nutritional, pharmaceutical, and energetic use, understanding of parameters that determine growth will become more and more important. However, modeling of plant growth is hampered by the dependence of resource allocation to either shoot or root on environmental parameters and the inability to record the root/shoot ratio non-invasively during the growth phase. To circumvent this problem, indirect parameters have been deployed to estimate the fraction of assimilates that are allocated to leaf growth and root formation, respectively. Models based on the transport-resistance model by Thornley^1^ focus on either shoot functions like starch metabolism^2^ and leaf area^3^ or root functions like water and nutrient uptake^4^ or sink strength.^5^ All these models handle biomass gain on a day-to-day basis, even though diurnal fluctuation of light intensity can be incorporated. Models based on “optimal partitioning”^6^ or on the “balanced growth hypothesis”^7^ are powerful at quantitatively describing the impact of environmental perturbations, but are insufficient at quantifying allocation for high-resolution in time. Poorter et al.^8^ demonstrated that even ontogenetic shifts in the root/shoot ratio could not be represented by these models. In contrast, modeling of metabolic dynamics is possible at hourly or even smaller time steps, thus restraining the simulation of the metabolic base of growth. Investigations of hourly resolved growth patterns of leaves in most cases rely on video capturing studies and have yielded diverging results. In the CAM intermediate *Clusia minor*, leaf growth peaked in the night when in C3 mode of photosynthesis, while it was higher during the day in CAM mode.^9^ In field grown wheat, growth was larger during the day,^10^ while for maize grown in climate chambers the pattern was less clear.^11^ In soybean and tobacco, leaf growth appeared to prevail in the dark period,^12,13^ while it was stronger during daytime in Arabidopsis.^14^ Mielewczik et al.^15^ found that leaf growth tightly correlated with air humidity, while it did not correlate with temperature. This reflects an important aspect of the imaging experiments: they document leaf expansion rather than structural carbon gain, and are thus strongly depending on the leaf water status. Growth is a complex phenomenon, integrating various metabolic pathways. Not only is the uptake of CO_2_ crucial for plant biomass production, but also sugar biosynthesis, carbon allocation to sink tissue, and respiration. Elucidation of the interactions of these pathways is crucial to understanding of the functions of the central metabolism for leaf biomass formation. Here, we present a stepwise development of a metabolic model that is capable of integrating different pathways (sugar metabolism, carbonic acid metabolism, amino acid synthesis) for simulating leaf structural carbon formation. The resulting dynamic model of the central metabolism of *A. thaliana* leaves can simulate shoot structural carbon formation at high resolution in time. This was achieved by allocating carbon, gained through photosynthesis, to either metabolic pools, root supply, or leaf structural carbon. The latter pool contains all carbon allocated to compounds with long residence time like cell wall or proteins. In this approach, allocation of carbon was independent of imaging data, as shoot structural carbon gain was calculated solely based on photosynthesis and metabolite data. To demonstrate applicability of the model under varying environmental conditions, simulations were performed for Arabidopsis plants under different light conditions.

Sugars are the primary product of photosynthetic carbon fixation, but are also key metabolites for regulation of primary metabolism.^16,17^ To investigate the importance of sugar signals for regulating shoot structural carbon formation, we included the hexokinase-1 null mutant, *gin2-1*, in the simulations. The *gin2-1* mutant is defective in the HXK1 enzyme activity and in glucose sensing, the mechanism of which is still not fully understood.^18^ However, sugar sensing and signaling is pivotal for modulating structural carbon formation, development, and stress responses.^19–22^ The *gin2-1* mutant is known to be high light sensitive and displays increasing growth retardation under rising light intensities.^23^ One important task was to set up a model that is able to represent the *gin2-1* phenotype.

## Results

### Photosynthesis and allocation of carbon to shoot and root

**Fig. 1:**
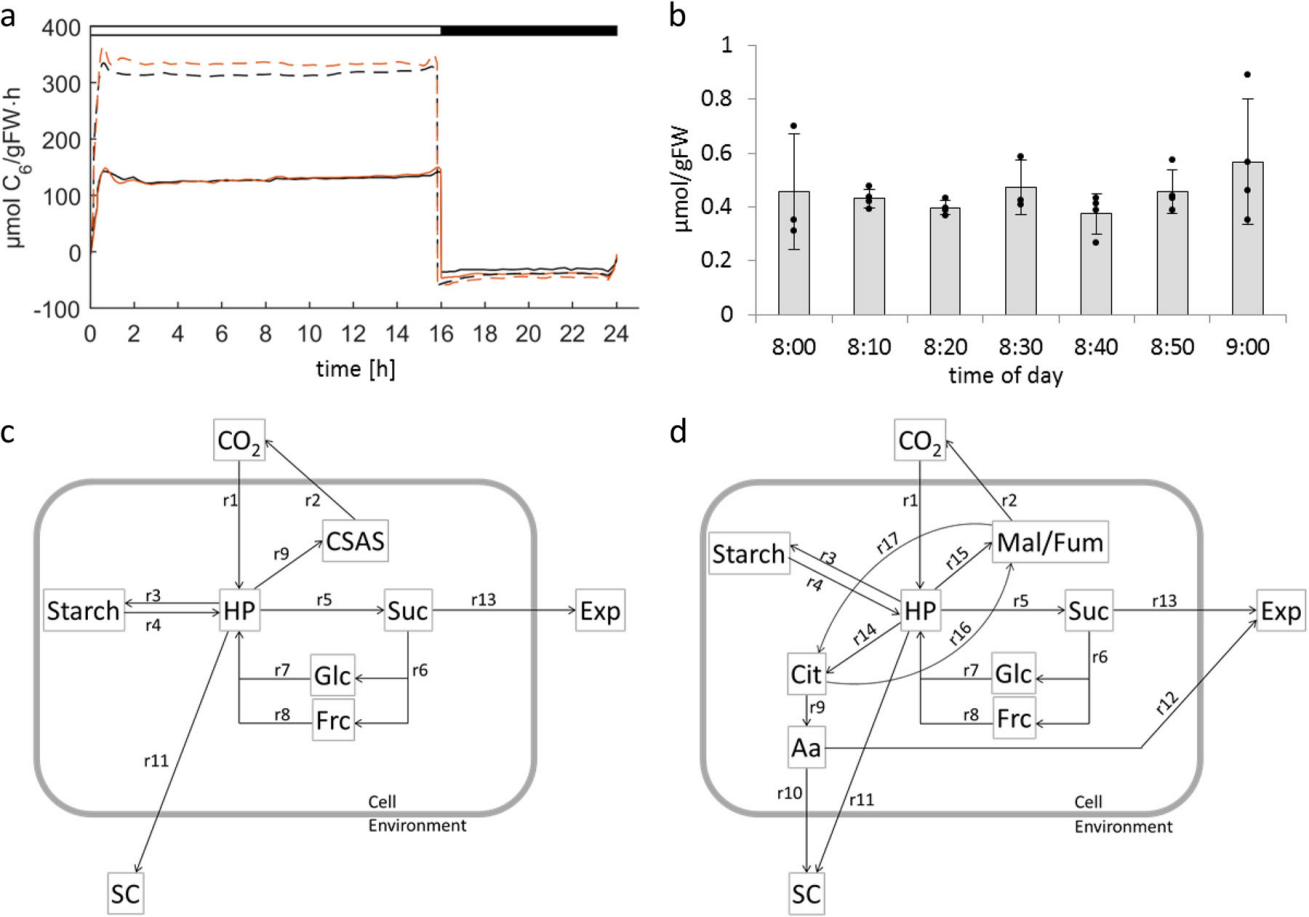
**a** Means of net photosynthesis rates of Ler (black) and *gin2-1* (orange) under normal (solid lines) and high light (dashed lines) conditions over a complete diurnal cycle (*n* = 9–13). Day and night are implicated with white and black bars on top. **b** Bar graphs of hexose phosphates (*n* = 3 or 4) within the first hour of the light phase, 8:00 to 9:00, under high light conditions in the *gin2-1* mutant. Individual data points (black dots) are layered over the respective bar. **c**, **d** Schematic topology of the simulated models for central metabolism with the basic model in panel **c** and the final, complex model in panel **d**. CaAa carbonic- and amino acids, HP hexose phosphates, Suc sucrose, Glc glucose, Frc fructose, Exp export, SC structural carbon (all compounds with long residence time like cell wall or proteins), Mal/Fum malate and fumarate, Cit citrate, Aa amino acids. For detailed information of the reaction rates (*r*1 to *r*17) see Table 4

Based on 9–13 independent CO_2_-exchange measurements over complete diurnal cycles (see Materials and methods) mean net photosynthesis (NPS) was calculated (µmol CO_2_ gFW^−1^ h^−^^1^). As shown in Fig. 1a, the CO_2_-fixation rate was approximately 130 µmol CO_2_ gFW^−^^1^ h^−1^ for both, Ler and *gin2-1* under control condition. CO_2_-fixation rates differed significantly (*p* < 0.005) between the high light and control condition. Under high light the rate increased almost 2.4-fold for Ler, reaching 310 µmol CO_2_ gFW^−1^ h^−^^1^ and 2.5-fold for *gin2-1*, reaching approximately 330 µmol CO_2_ gFW^−1^ h^−^^1^. Shoot respiration was not significantly affected by either genotype or condition during the night, reaching on average 28 µmol CO_2_ gFW^−1^ h^−^^1^ for Ler and 37 µmol CO_2_ gFW^−1^ h^−^^1^ for *gin2-1* under the control condition and about 45 µmol CO_2_ gFW^−1^ h^−^^1^ for Ler and *gin2-1* under the high light condition. Minor deflections around the day/night transition resulted from fitting curves to the measured data. The daily structural carbon gain of shoot tissue was calculated from the daily increment of net photosynthesis (see Fig. 1a) as described in Materials and methods (Eq. 1), amounting to 120 µmol C_6_ gFW^−1^ for Ler control, 100 µmol C_6_ gFW^−1^ for *gin2-1* at control, and 135 µmol C_6_ gFW^−1^ for Ler but only 85 µmol C_6_ gFW^−1^ for *gin2-1* at high light. The data revealed a large discrepancy between the ratio of photosynthesis and shoot structural carbon gain for Ler and *gin2-1* especially under high light, which could have resulted from either increased assimilate export to sink organs or build-up of excess carbon storage pools. To test the latter possibility, quantitatively relevant metabolite pools were analyzed.

### Central carbon metabolism

As quantitatively relevant central metabolites, starch, hexose phosphates (HP), glucose (Glc), fructose (Frc), sucrose (Suc), amino acids (Aa), malate (Mal), fumarate (Fum), and citrate (Cit) were determined in 2 h intervals over a full diurnal cycle (Fig. S9). After one diurnal cycle under control conditions each pool returned to the starting value. However, after one day in high light, the metabolite values are significantly higher, except for citrate. The data represent means for six independent complete *A. thaliana* rosettes exposed to either normal or high light condition. Significant effects of light condition or genotype are listed in Table 1. Main differences between genotypes occurred for soluble sugars and malate under both conditions and for citrate and amino acids for the control condition only. Only citrate was lower in Ler as compared to *gin2-1*, and thus Ler displayed a larger pool of fixed carbon. Almost all metabolite concentrations increased under high light in both genotypes, again with the exception of citrate. Starch and soluble sugars rose 3- to 5-fold and carboxylic and amino acids 1.5- to 3-fold throughout the whole diurnal cycle, while hexose phosphates rose 1.5- to 2-fold only during the night.

**Table 1:** Comparison of means for central carbon metabolites over a 24 h cycle for either the genotypes (Ler and *gin2-1*), or the light regime (high light and control condition).

	N	HL	Ler	*gin2-1*
	Ler:*gin2-1*	Ler:*gin2-1*	HL:N	HL:N
Sta	n.s.	n.s.	***	***
HP	n.s.	n.s.	**	**
Glc	***	***	***	***
Frc	***	***	***	***
Suc	***	***	***	***
Mal	***	***	**	***
Fum	n.s.	n.s.	***	***
Cit	*	n.s.	n.s.	n.s.
Aa	***	n.s.	***	***

*Sta* starch, *HP* hexose phosphates, *Glc* glucose, *Frc* fructose, *Suc* sucrose, *Mal* malate, *Fum* fumarate, *Cit* citrate, *Aa* amino acids, *N* control condition, *HL* high light condition (***p < 0.0005, **p < 0.005, *p < 0.05, analysis of variance, Tukey HSD, *n.s.* not significant)

As for metabolites, the maximal reaction rates of the enzymes participating in sucrose cycling^24,25^ were determined at saturating substrate concentration in 2 h intervals over a full diurnal cycle (Fig. S10). Since no subcellular compartmentation was implemented in the model, activity of acidic and neutral invertase (Inv) were measured separately, but then summed up as one combined maximal reaction rate to represent the whole sucrolytic capacity of the leaf tissue. As expected, significant differences were observed for hexokinase activity (glucokinase and fructokinase) between Ler and *gin2-1* under control as well as high light conditions. Surprisingly the fructokinase activity was lower in high light as compared to control condition in Ler. In addition, significant differences were found for sucrose-phosphate-synthase (SPS) activity, which was almost two-fold higher under high light for both genotypes. The Inv activity was higher in *gin2-1* compared to Ler under both conditions.

### Modeling

The metabolic model focused on the plant aerial organs as a sole source for carbon fixation. All carbon that was not contained in one of the metabolite pools was considered to either contribute to structural carbon formation in the source tissue or be exported to sink organs, which, in the chosen experimental setup, are represented solely by the root system. Exported assimilates are either used for root respiration or used root structural carbon formation. This was not further resolved, because root material was not accessible in soil grown plants. Model development started from a simple model as introduced by Nägele et al.^24^ This model comprised NPS as input, four carbohydrates, hexose phosphates as central hub, one combined pool of carboxylic and amino acids (CaAa), and three possible outputs: (i) shoot structural carbon gain, (ii) export of sucrose to sink organs and (iii) respiratory release of CO_2_ from CaAa during the night (Fig. 1c). The final model shown in Fig. 1d was built up in seven steps, each increasing the complexity by adding regulatory and/or metabolic details, which are listed in Table 2. The final model contained several branch points, two cycles and various regulatory terms affecting enzyme activities. The two cycles are: (i) the sucrose cycle, in which HP are used as substrate by SPS to produce sucrose, which is then degraded by Inv to glucose and fructose, each of which can be re-phosphorylated to HP by hexokinase (Hxk), and (ii) the tricarboxylic acid cycle (TCA). The last step of sucrose cycling is restricted in the *gin2-1* mutant as *gin2-1* does not possess the HXK1 enzyme. This resulted in five- to seven-fold reduction in sucrose cycling in the mutant as calculated from the flux through *r*6 (Fig. S8).

**Table 2:** List of the different models, their variation compared to the previous model, and the outcome or idea for the next model

Model no.	Variation compared to previous model	Features/limitations	Required improvements
01	Basic model	Strong HP deflection at day/night transition	Better map day/night transition
02	Adjusted starch synthesis and degradation (*r*3 and r*4*)	Sharp deviance of CaAa pool at day/night transition	Introduce a constant respiration rate
03	Constant respiration rate during day and night		Split carbon pools to converge closer to the in vivo condition
04	Split carbon pool into a citrate and combined malate/fumarate pool	increased dynamics for Cit and MalFum	Interconversion of Cit and MalFum as in the TCA cycle
05	Interconversion between Cit and MalFum	Long simulation times (up to 8 h per simulation)	Splining of enzyme parameters to reduce simulation time
06	Splines for *V*_m_ values for sucrose cycling enzymes	Flat dynamics for Glc and Frc	Splining of selected enzyme parameters
07	Spline for SPS and Inv	Shorter simulation times (up to 4 h per simulation)	Exact distribution of excess carbon to shoot or root
08	Implementing the calculated SC gain	Final model	

*HP* hexose phosphates, *CaAa* carbonic- and amino acids, *Cit* citrate, *MalFum* malate and fumarate pool, *Glc* glucose, *Frc* fructose, *SPS* sucrose-phosphate-synthase, *Inv* invertase, *SC* structural carbon

Sole input for the final model is still CO_2_ fixation, but the output of the system has become more complex, as amino acids are now able to leave the system through either structural carbon formation in the source tissue or export, and respiration of CO_2_ is now only possible from the Mal/Fum pool. The Cit pool is linked to the Aa and Mal/Fum pool through interconversion terms and does therefore contribute indirectly to the output of the system. During the night photosynthesis is stalled, and carbon used for respiration, metabolite interconversions, or sink export must originate from starch degradation. Again, HPs are the central hub for metabolite interconversions. Although the HP concentration is in the sub-micromolar range, the simulated flux through this pool was found to be very high, as all carbon will be fixed in the form of HP (Fig. 1c, d) and thus, the highest fluxes, CO_2_-fixation and starch synthesis/degradation, will contribute to flux through the HP pool. Almost every model showed an HP peak at the beginning of the light phase, especially for *gin2-1* models. To test if this was real, we sampled *gin2-1* plants every 10 min during the first hour after light-on under high light condition to maximize the light effect and analyzed HP content. We were unable to confirm this peak in the samples (Fig. 1b). We therefore conclude that it is an artifact, probably arising from the on/off behavior of the CO_2_-fixation and the 2-h time resolution of the metabolic profile used for model simulations.

### Model characteristics

**Fig. 2:**
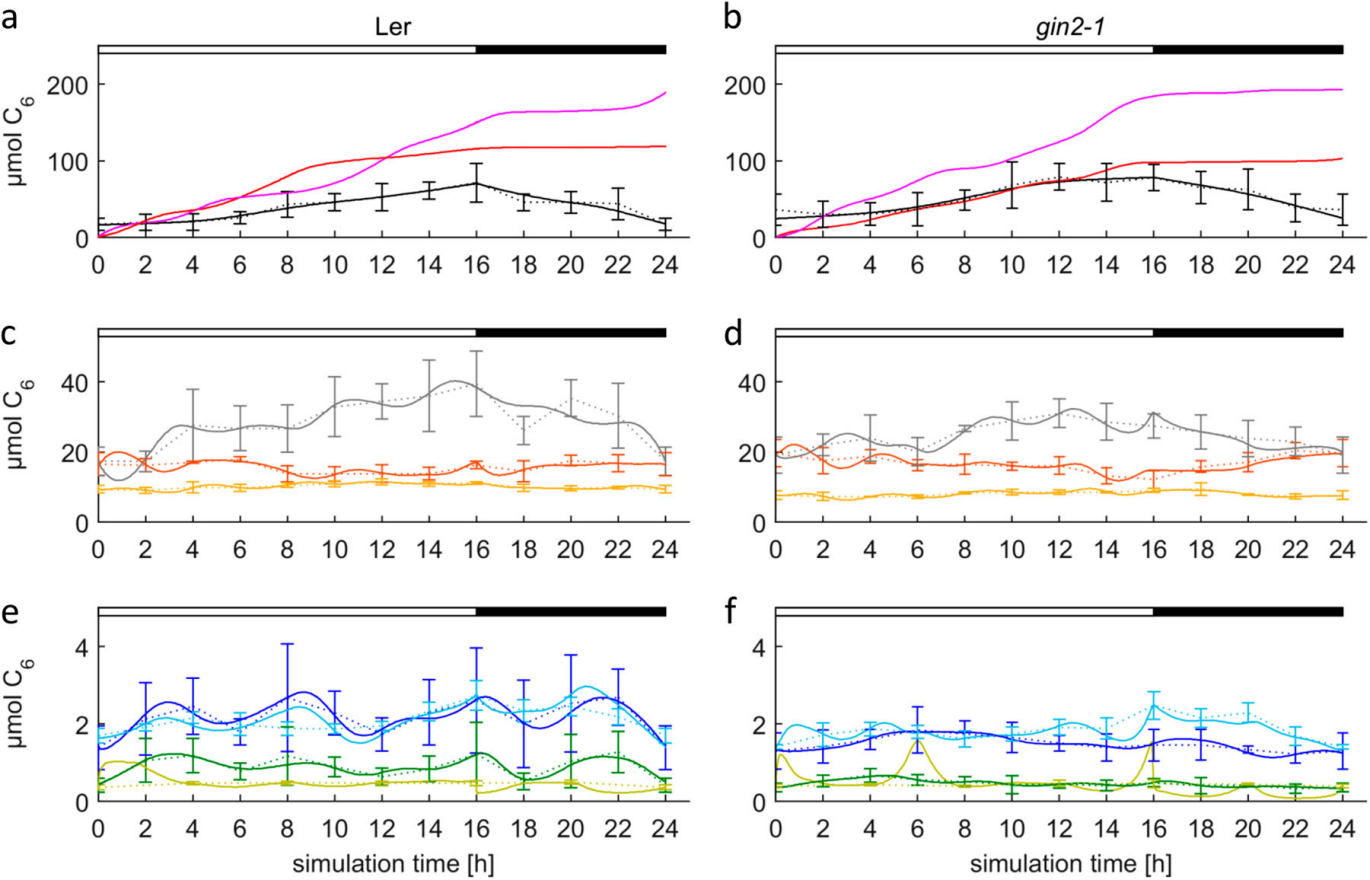
Simulated results for the final Model_ 08 under control condition. Dotted lines represent means of measured data ± SD (*n* = 6), solid lines represent means of model simulations (*n* = 5) for Ler (**a**, **c**, **e**) and *gin2-1* (**b**, **d**, **f**). Day and night are indicated with white and black bars on top. Sucrose is expressed as C_12_. **a**, **b** starch (black), structural carbon (red), export (pink). **c**, **d** malate/fumarate pool (gray), citrate (orange), and amino acids (yellow). **e**,**f** glucose (blue), sucrose (turquois), fructose (green), and hexose phosphates (olive)

**Fig. 3:**
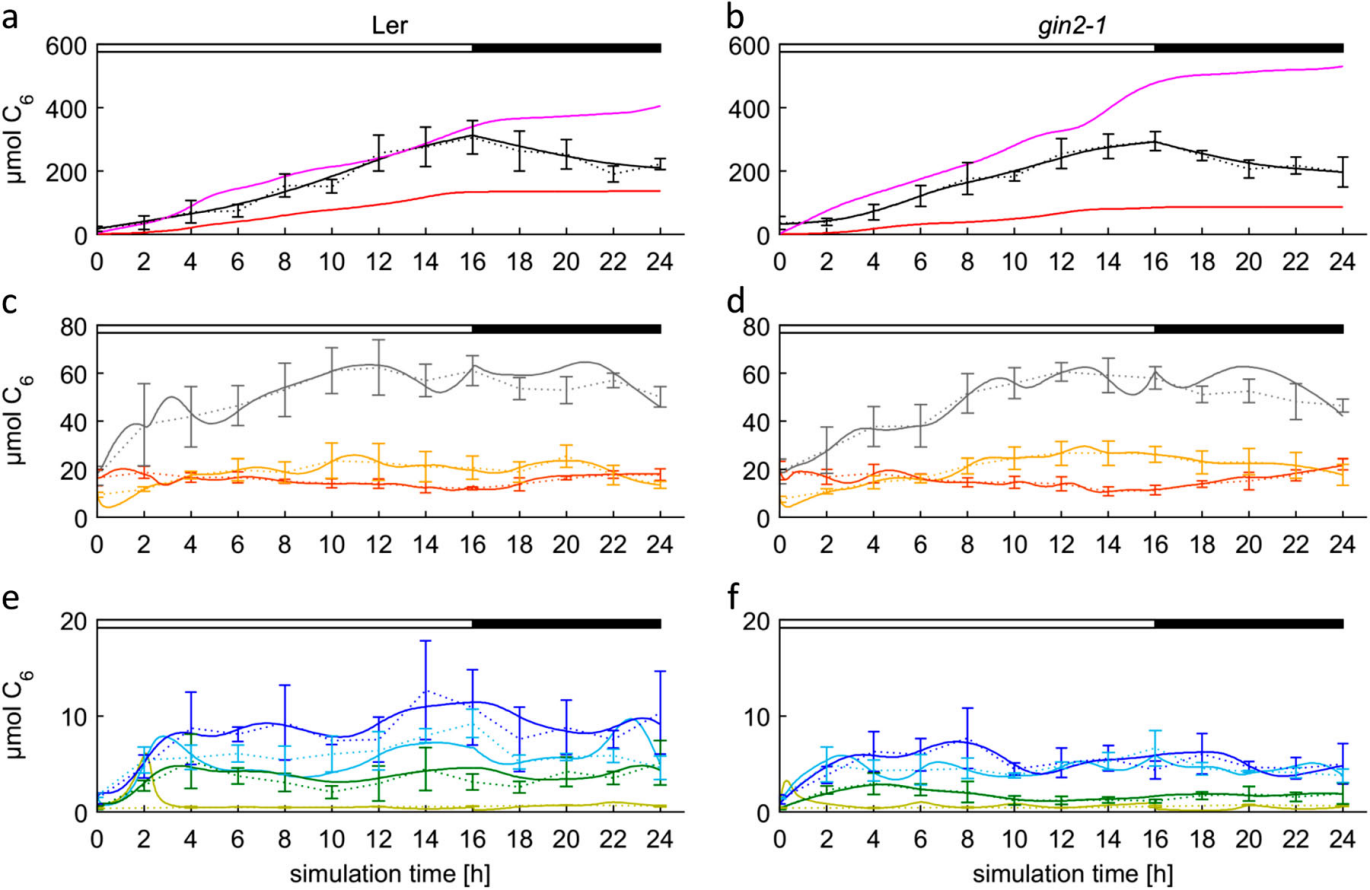
Simulated results for the final Model_08 under high light condition. Dotted lines represent means of measured data ± SD (*n* = 6), solid lines represent means of model simulations (*n* = 5) for Ler (**a**, **c**, **e**) and *gin2-1* (**b**, **d**, **f**). Day and night are indicated with white and black bars on top. Sucrose is expressed as C_12_. **a**, **b** Starch (black), structural carbon (red), export (pink). **c**, **d** Malate/fumarate pool (gray), citrate (orange), and amino acids (yellow). **e**, **f** Glucose (blue), sucrose (turquois), fructose (green), and hexose phosphates (olive)

The final model was developed in seven steps as listed in Table 2. Each of these steps addressed a limitation of the previous model, thus allowing to refine understanding of essential features of the metabolic system. For example, a strict switch between starch synthesis and degradation was necessary to prevent overflow of the hexose-phosphate pool (Model 01 vs. 02, compare Figures S1 and S2), a constant respiratory activity over the full diurnal cycle was necessary to prevent deflections in the pool of carbonic and amino acids (Model 02 vs. 03, compare Figures S2 and S3), and the possibility of interconverting Cit and Mal/Fum was needed to faithfully map levels of carbonic acids (Model 04 vs. 05, compare Figures S4 and S5). A more detailed explanation of each modeling step is given in the supplementary material (Text_S1, “Model characteristics”). Despite increasing complexity, the model retained its accuracy, as the values for the cost function did not rise to values higher 0.018 (Table 3), while representation of the in vivo situation became more exact. The final model was used to simulate the dataset for the high light conditions without additional modifications, thus underlining the general validity of the approach. Under normal light conditions (Fig. 2), the models were able to accurately predict concentrations of most metabolites, except for HP in *gin2-1*. Here, three artificial peaks occurred: the first right after light-on, a second at 6 h into the light, and the third at the day/night transition. Both genotypes displayed steady gain of structural carbon during the day, but not during the night. This is not an obvious outcome, since carbon flux from metabolite degradation (e.g. starch) is higher than the respiration rate. This excess carbon could have been allocated to shoot structural carbon formation in the night. However, in optimal simulations, the excess carbon was allocated to the sink tissue. Sink export occurred throughout the whole diurnal cycle for Ler under both conditions and for *gin2-1* under high light. However, mean export rates were 2–2.5-fold higher for Ler (day, night control: 9.4 C_6_/h, 4.8 C_6_/h; day, night high light: 21 C_6_/h, 8 C_6_/h) and 5- to 10-fold higher for *gin2-*1 (day/night control: 11.5 C_6_/h, 1.1 C_6_/h; day/night high light: 30 C_6_/h, 6.5 C_6_/h) during the day than during the night, and the export rates for *gin2-1* during the day were generally 1.2- to 1.5-fold higher than the export rates for Ler. Although *gin2-1* had a low sink export rate during the night under control conditions, export still sufficed for roots maintenance metabolism. The higher sink export in *gin2-1* resulted in an equal reduction in shoot structural carbon formation. This points to an important role of hexokinase-1 in carbon allocation by coordinating sucrose cycling and sugar sensing. Simulations of the final model for high light are shown in Fig. 3. Minor miss-alignments were observed for Mal/Fum in Ler and g*in2-1* and for Frc in Ler. Except for soluble sugars, only small differences in metabolite concentrations between the two genotypes were observed. However, large deviations occurred for structural carbon. For both genotypes, the curve of structural carbon gain showed constant rise for the source tissue during the light phase with a delay of 6 h at dawn in Ler that was absent in *gin2-1*. Shoot structural carbon gain was not more than 3 µmol C_6_ gFW^−^^1^ during the night and therefore negligible for both genotypes and conditions.

**Table 3:** List of the cost function (sum of squared errors between the measured and simulated data points) for each step after model optimization (*n* = 5 ± SD)

Model no.	Ler	*gin2-1*
01	0.0055 ± 0.00015	0.0026 ± 0.00044
02	0.0101 ± 0.00023	0.0043 ± 0.0011
03	0.0136 ± 0.0022	0.0051 ± 0.00035
04	0.0072 ± 0.00053	0.0101 ± 0.00033
05	0.0075 ± 0.00033	0.0021 ± 0.0001
06	0.0136 ± 0.00031	0.0061 ± 0.0012
07	0.0074 ± 0.00038	0.0018 ± 0
08 N	0.0059 ± 0.000055	0.0019 ± 0.00015
08 HL	0.0179 ± 0.005	0.0067 ± 0.000045

## Discussion

Plant growth represents the integrated output of numerous molecular processes which are involved in photosynthetic CO_2_ uptake, sugar biosynthesis, carbon allocation, and mitochondrial respiration. To link metabolic regulation in central primary metabolism of Arabidopsis with growth processes, we developed a dynamic mathematical model to simulate structural carbon allocation to shoot tissue under varying environmental conditions. In previous studies, we have observed non-intuitive coherences between metabolic enzymes, carbon allocation and growth.^24,26^ Thus, extending previous work, the main goal of the present study was to develop a kinetic model allowing to simulate the interaction of central leaf carbon metabolism and structural carbon formation during a full diurnal cycle in order to yield a mechanistic understanding of how carbon allocation is affected by metabolic regulation. Thus, we compared Ler to its HXK1 mutant *gin2-1*, for which a growth retardation at light intensities above 160 µmol m^−^^2^ s^−^^1^ is well documented,^23^ and found that although *gin2-1* increased its carbon fixation rate even 2.5-fold under high light, the structural carbon gain of the leaf rosette was even 15% smaller than under normal light, while it increased by 13% in Ler.

With non-limiting water and CO_2_ supply, net photosynthesis is expected to increase with illumination intensity until it reaches an optimum, at which further increase in light intensity may cause damage to photosystems. We indeed observed a 2.4- to 2.5-fold increase in carbon fixation rate for Ler and *gin2-1*, when the light intensity was raised from 120 to 1200 µmol m^−^^2^ s^−1^. Although photosynthetic CO_2_ fixation did increase by the same ratio in both genotypes, leaf structural carbon formation behaved, as expected, in an opposite way: it increased in Ler but decreased in *gin2-1*. This indicated that the absence of sugar sensing and/or a reduced sucrose cycling had a strong impact on carbon allocation. Brauner et al.^26^ reported that higher photosynthetic efficiency of *gin2-1* did not translate into a higher growth rate because of elevated assimilate export to the root system, where respiratory activity was increased. Elevated sugar levels were discussed as the cause for increased export, but data presented here offer an alternative possibility. According to Meyer et al.^27^ high growth rates correlate with a metabolic signature comprising low levels of central metabolites like hexose phosphates, sucrose, and carboxylates, thus indicating that fast growth drains the pools of central metabolites. Comparing Ler and the *gin2-1* mutant, we detected lower levels of sugars and malate in *gin2-1* under high light conditions, while hexose phosphates remained unchanged (see Table 1 and Fig. S9). Thus, insufficient build-up of central metabolites could be the cause of slower growth, and elevated sugar export might be its consequence. This would point to the inability of *gin2-1* to sense the sugar content being the cause of slow growth.

To sort this out, we developed a dynamic metabolic model that simulates the diurnal course of structural carbon formation in the source tissue and C-export, as a function of net photosynthesis and dynamics of metabolite pools. Because structural carbon gain of the shoot and assimilate export from leaves to the root are competing output terms in the model, it was necessary to set a margin for at least one of the two. For daily shoot structural carbon gain this was achieved by calculating the increment of total net photosynthesis (NPS) within 24 h assuming that specific leaf area (SLA) did not change within 24 h, the gain in specific NPS (NPS/gFW) would then reflect extension of the leaf rosette and therefore leaf growth (Eq. (1), see Materials and methods). This concept is supported by Tocquin et al.^28^ who found that SLA, although depending on atmospheric CO_2_ concentration, N-supply, and plant age, remained remarkably constant in adult Arabidopsis plants during vegetative growth.

With a limit set to the proportion of photosynthate allocated to shoot structural carbon, a ratio of carbon use for root and shoot of 0.5 to 0.6 was obtained. Considering the high respiratory activity of the root, this is in agreement with the root/shoot ratio reported for Arabidopsis,^26^ thus supporting our concept. All model simulations placed leaf structural carbon formation into the light phase with almost constant rates that declined within the last 2 h of the light phase, except for Ler under control condition, where structural carbon gain ceased already 6 h before the night. This reflects imaging data obtained by Wiese et al.^14^ who reported leaf expansion predominantly in the early morning. As stated above, leaf expansion and biomass deposition are not interchangeable. Nevertheless, simulations indicate that for Arabidopsis these phenomena might be linked.

Although about one-fifth (15%) of the photosynthetic activity was used to build up starch during the light phase under normal light, and about 30–35% under high light, not only structural carbon formation but also assimilate export were strongly decreased during the night in model simulations. It thus appears that night-time metabolism in Arabidopsis may be predominantly dedicated to maintenance. This is different, for example, in potato, were similar sugar transport and growth rates of tubers have been reported for the light and dark phase.^29^ In the controversial discussion of day or night growth of plant organs, Pantin et al.^30,31^ have demonstrated that leaf expansion is limited hydromechanically in Arabidopsis already 4 days after leaf emergence under moderate air and soil humidity. Under these conditions, carbohydrates serve, at least in part, to release the hydromechanical limitation of leaf expansion, which, under water deficit, is shifted to the night-time. This fully agrees with our findings that place structural carbon deposition within the light period independent of the diurnal profile of leaf expansion.

### High light treatment

Shifting plants from a moderate light intensity (120 µmol m^−^^2^ s^−^^1^) to high light altered the photosynthetic input and metabolite pool sizes, and thus the model parameters. These alterations could still be simulated without structural changes in the model, proving its general applicability. Simulations could reproduce the deflection that was observed in the glucose-to-sucrose ratio especially in Ler as well as that in the ratio of citrate-to-amino acids that occurred in both genotypes. Although the increase in the starch pool led to higher assimilate transport rates during the night, structural carbon gain was still more or less confined to the light period. As expected, the growth retardation of the *gin2-1* mutant became more pronounced under high light. While structural carbon deposition in the mutant was about 80% of the wild type in moderate light, it even decreased under high light and reached only 64% of the wild-type level. Similar observations have been made by Moore et al.^23^ In *gin2-1* this caused the assimilate export rate to rise strongly, which is in agreement with the higher root respiration rate of this genotype.^26^ The reduced structural carbon deposition in *gin2-1* under high light (85% *gin2-1* under control condition) correlated with lower citrate levels, while all other metabolites accumulated. A correlation of low citrate with reduced growth was also reported for the phospho*enol*pyruvate carboxylase double-mutant *ppc1/ppc2* of Arabidopsis.^32^ Thus, suggesting a fundamental role of citrate for regulating growth. In the *ppc1/ppc2* mutant, the amino acids aspartate, asparagine, and glutamate were also reduced. Although we did not discriminate individual amino acids, it is unlikely that these amino acids were reduced in *gin2-1* considering that the entire pool doubled under high light and is to a very large extent dominated by these amino acids in Arabidopsis.^33^ The diurnal profile of citrate, which is opposite to the other primary metabolites, would fit with our finding of structural carbon formation during the light phase, reaching its lowest level at light-off, when growth ceases.

### Model development

We increased model complexity stepwise to better map the in vivo situation of the metabolic system. Although an increasing number of kinetic model parameters lead to an expansion of parameter space, optimization yielded a similar cost function. The calculated cost function (see Materials and methods) remained in a range between 0.002 and 0.018 (Table 3), indicating that model accuracy remained high (Fig. 1c, d). However, for the high light condition the cost function increased almost three-fold to 0.018 for Ler and 0.0067 for *gin2-1*. It must be considered that the calculated cost function is only giving the sum of squared deviations between simulated and measured data. This in turn means that a large error in metabolites with low abundance, such as HP and sugars, will contribute only very little to the cost function, while small errors in metabolites that are highly abundant, like starch, are contributing eminently to the cost function. The three-fold increase of the cost function value for the high light simulation is therefore due to the higher concentrations for almost all metabolites included in the model. It is also important to keep in mind that the cost function can only calculate residuals, when a measured data point is available. Extreme oscillations of the simulated data between two data points would therefore not affect the cost function value as long as the function meets the measured metabolite concentrations. Judging the goodness of a model solely based on the cost function values might therefore lead to misinterpretations. Thus, additional control of the model output by plotting the simulated and the measured data is necessary in any case.

The stepwise construction of the metabolic model presented in this study revealed interesting details of metabolic interactions, which would not have been detected without a stepwise analysis and comparison of the different models: A simple model with merged metabolite blocks (carboxylates and amino acids, starch, various sugars) was able to prove consistency of the measured data for photosynthesis and metabolite interconversions. It also showed that the day/night transition must involve a sudden change from a starch synthesis to a starch degradation mode to prevent an overflow of the HP pool. As starch is one of the most abundant metabolites in plants, regulation of its synthesis and degradation is of enormous interest. Many articles and reviews deal with the question how starch synthesis and degradation is regulated.

This is supported by diverse models describing circadian control of starch turnover.^34–37^

For example the model of Seaton et al.^35^ comprised a simple differential equation for starch metabolism that is a function of light availability, and thus can only assume the values 1 or 0. The equation will therefore either give starch synthesis or degradation. With this equation Seaton et al. were able to explain all environmental perturbations of starch metabolism tested. In contrast, Feugier and Satake^34^ demonstrated that, when the sucrose pool is modeled as starch degradation product, sucrose runs over, when starch degradation occurs during the light phase. In our model, the starch degradation product is HP, and simulations based on a steady transition between starch synthesis and degradation showed similar results, emphasizing the importance of this step in model development. HP accumulation turned out to be stronger in the *gin2-1* mutant as compared to the wild type. Although this might not be expected considering the lack of glucokinase activity, it has already been observed by Moore et al.^23^ The accumulation of glucose-6-phosphate could further reduce sucrose cycling in the *gin2-1* mutant by inhibition of invertase, thus aggravating the effect of the mutation. Besides starch metabolism, the mode of respiration is as well highly discussed in the literature.^38–40^ It is experimentally very difficult to measure photosynthesis and respiration simultaneously. Although it is generally accepted that mitochondrial respiration is needed to provide C skeletons for amino acid synthesis,^38^ the question as to what extend respiration takes place in an illuminated leaf is under debate. In vivo measurements in French bean^39^ did not support respiration during the day, while measurements in maize leaves^40^ revealed the opposite. A constant respiration activity of leaf cells throughout the diurnal cycle based on the measured dark respiration improved simulation of the combined pool of carboxylates and amino acids in both genotypes, while it had little effect on carbohydrates. Our model simulations are therefore in support of a constant respiratory activity, which removed the kink in the simulated combined CaAa pool that occurred at day/night transition, when respiration was allowed only during the dark phase.

Continuous respiration creates a constant flux through the TCA cycle and thus afforded separation of the combined CaAa pool into carbonic and amino acids. The CaAa pool was initially intended to keep “non-carbohydrate” stores of assimilated carbon. By isotopic labeling, it was demonstrated that, instead of running as a true cycle, the TCA pathway splits into two separate branches in the light, one providing oxoglutarate using stored citrate as a substrate and one producing malate and fumarate via phosphoenolpyruvate carboxylase (PEPC) thus inverting the regular mode of operation.^41^ As a consequence, amino acid synthesis relies mainly on C skeletons stored during the previous day and is largely independent of actual photosynthesis.^42,43^ Interestingly, model simulations showed that complete separation of the branches is unfounded. Only after introduction of a “regular” TCA cycle like conversion between Mal/Fum and Cit, dynamics were simulated correctly. This shows that the anaplerotic function of PEPC to produce citrate via oxaloacetate is indispensable for amino acid synthesis during the day, as claimed by Tcherkez et al.^41^ and Nunes-Nesi et al.^44^ This also agrees with a diurnal flux balance analysis that, in contrast to a continuous light model, predicted citrate production via the mitochondrial TCA cycle and not by peroxisomal citrate synthase.^43^

To reduce the number of parameters which needed to be estimated, and therefore reduce the simulation time, we decided to provide the maximal reaction rates for enzymatic reactions describing sucrose cycling: SPS, Inv, and Hxk (gluco- and fructokinase) as smoothing splines through the measured values. Unexpectedly, splining all four maximal reaction rates drastically decreased the glucose and fructose dynamics, especially visible in the *gin2-1*, thus masking the phenotype of the mutant (Fig. S6D). Therefore, only the reaction rates of SPS and Inv were implemented as smoothing splines in the final model. When the model was allowed to allocate excess carbon freely between shoot structural carbon and export to sink organs, plant growth could not be simulated reliably. By implementing the calculated daily structural carbon gain (Eq. (1)) into the model, we forced adequate allocation of excess carbon to shoot structural carbon formation, thus resulting in correct allocation of carbon between the source tissue and assimilate export to sink organs. The simulated formation of structural carbon showed the characteristics of a typical saturation curve, with a positive slope during the day that became zero around day/night transition. This detailed model for the central metabolism of *A. thaliana* can be further used for studies on growth, carbon allocation, or the metabolic behavior of *A. thaliana* under different environmental conditions. It might also be possible to refine the model by increasing the time resolution around the day/night transition or expanding the model by adding, for example, the pentose phosphate pathway or the Calvin–Benson cycle.

## Materials and methods

### Plant material and growth conditions

*A. thaliana* (L.) Heynh., accession Landsberg *erecta* (Ler) and the *gin2-1* knockout mutant of hexokinase-1 (At4g29130), were grown in a 1:1 mixture of GS90 soil and vermiculite in a growth chamber with a 8 h/16 h light/dark regime (120 μmol m^−2^ s^−^^1^; 22 °C/16 °C). After 5 weeks, plants were transferred to long day and a 16 h/8 h light/dark regime was applied (120 μmol m^−^^2^ s^−^^1^; 22 °C/16 °C). The relative humidity was 70%. Plants were watered regularly and fertilized with standard nitrogen–phosphate–potassium fertilizer immediately after pricking, ten days before and four days after transfer to long-day condition. Eight days after transfer to long-day condition the plants were harvested every 2 h for a full 24 h diurnal cycle, starting immediately before the light-on phase. One half of the plants were harvested under normal growth conditions and the other half was exposed to high light (1200 μmol m^−2^ s^−^^1^) for 16 h and was harvested within the same time frame (every 2 h for 24 h). For metabolite and enzyme analysis, whole rosettes of ten independent biological replicates were sampled for each time point, condition, and genotype. For the *gin2-1* mutant two rosettes were pooled for one independent sample. Samples were immediately frozen in liquid nitrogen, frozen leafs were ground to a fine powder using a MM200 ball mill (Retsch, Retsch GmbH, www.retsch.de) and stored at −80 °C until further use.

### Metabolite analysis

For soluble sugars and starch, pulverized plant material was extracted twice in 400 µl of 80% ethanol at 80 °C. Extracts were dried and dissolved in 500 µl of distilled water. Contents of glucose, fructose, and sucrose were analyzed by high-performance anion-exchange chromatography (HPAEC) using a CarboPac PA-1 column on a Dionex (Sunnyvale, CA, USA) DX-500 gradient chromatography system coupled with pulsed amperometric detection by a gold electrode. For starch extraction, pellets of the ethanol extraction were solubilized by heating them to 95 °C in 0.5 N NaOH for 45 min. After acidification with 1 N CH_3_COOH the suspension was digested for 2 h with amyloglucosidase. The glucose content of the supernatant was indirectly determined photometrically at a wavelength of 540 nm with o-dianisidine and used to assess the starch content of the sample.

The hexose-phosphates glucose-6-phosphate and fructose-6-phosphate were measured as described previously,^45^ volumes were downscaled for analysis in a 96-well plate and were measured in a plate reader at 570 nm (TECAN-SpectrafluorPlus, Männedorf, Switzerland).

The content of the carbonic acids malic acid, fumaric acid, and citric acid were determined by anion-exchange chromatography on a AS11-HC column (Dionex, Sunnyvale, CA, USA) using a gradient from 10 to 20 mM NaOH running on a DX-500 gradient chromatography system coupled to an AERS 500 suppressor and a conductivity cell (Dionex, Sunnyvale, CA, USA).

Amino acids were measured by a colorimetric ninhydrin assay at a wavelength of 570 nm. Frozen plant material was extracted in a similar way as the soluble sugars. After the extraction steps with ethanol, the pellet was further extracted with 500 µl H_2_O. Ethanol fractions were dried and the water extract was used to dissolve the dried ethanol fraction.

### Measurement of enzyme activities

Enzyme activities were determined in crude extracts of pulverized plant material. All measurements of enzyme activity took place at vast substrate excess,^25,46,47^ yielding a nonstandard estimate of the maximum reaction rate (*V*_m_) for the enzymes under our conditions. Maximal reaction rate of acid invertase and neutral invertase was determined in frozen leaf tissue, homogenized in 50 mM Hepes-KOH (pH 7.4), 5 mM MgCl_2_, 1 mM EDTA, 1 mM EGTA, 1 mM phenyl-methyl-sulfonyl-fluoride (PMSF), 0.1% Triton X-100, and 10% glycerol. The suspension was centrifuged at 13000 *g* for 5 min at 4 °C. Soluble acid invertase was assayed in 20 mM Na-Acetate buffer (pH 4.7) using 100 mM sucrose as a substrate. Neutral invertase was assayed in 20 mM Hepes-KOH (pH 7.5) using 100 mM sucrose as a substrate. The control of each assay was boiled immediately for 5 min. Reactions were incubated for 30 min at 30 °C, stopped by boiling for 5 min. The concentration of glucose was indirectly determined photometrically at a wavelength of 540 nm with o-dianisidine. Activity of glucokinase and fructokinase was measured as described in Wiese et al.^46^ Synthesized glucose-6-phosphate was converted to 6-phosphogluconolactone by glucose-6-phosphate-dehydrogenase and could be measured photometrically at a wavelength of 340 nm as a change in concentration of the reduced co-substrate NADPH. For isomerization of fructose-6-phosphate, phosphoglucoisomerase was added.

Activity of sucrose-phosphate-synthase (SPS) was determined in homogenates of frozen leaf tissue in 50 mM Hepes/KOH (pH 7.5), 10 mM MgCl_2_, 1 mM EDTA, 2.5 mM dithiothreitol (DTT), 10% glycerol and 0.1% Triton X-100. Suspensions were centrifuged at 13000 *g* and 4 °C for 5 min. SPS activity was assayed in supernatants as described previously^24^ shortly: The supernatants were incubated with 50 mM Hepes/KOH (pH 7.5), 15 mM MgCl_2_, 2.5 mM DTT, 35 mM UDP-glucose, 35 mM fructose-6-phosphate, and 140 mM glucose-6-phosphate for 4, 10, and 20 min at 25 °C. The reaction was stopped by boiling it for 10 min after the addition of 30% (w/v) KOH. This mixture was then measured, after incubation with 0.14% (w/v) Anthron in 14.6 M H_2_SO_4_ for 30 min at 40 °C in a water bath, photometrical at a wavelength of 620 nm in cuvettes preheated at 40 °C.

### Gas exchange measurement

The exchange rates of CO_2_ were measured using an infrared gas analysis system (Uras 3G; Hartmann & Braun AG, Frankfurt am Main, Germany). A whole-rosette cuvette design was used as described in Nägele et al.^24^ Gas exchange was measured in the growth chamber for 24 h under normal and high light conditions. Means of raw data for gas exchange were converted to flux rates per gram fresh weight (gFW) obtained at the end of the measurement by weighing complete rosettes. The experiment was repeated 9–13 times per condition and genotype.

### Structural carbon formation

Specific structural carbon formation of the shoot for one day was calculated after measuring photosynthetic activities. Based on the assumption that growth of leaves will increase photosynthesis,^2,48^ the following equation was defined to calculate leaf structural carbon gain:

1 $$\mathop {\int }\limits_{t1}^{t2} {\mathrm {rPS1}}{\mathrm d}t/{\mathrm {gFW1}} = \mathop {\int }\limits_{t1}^{t2} {\mathrm {rPS2dt}}/{\mathrm {gFW2}}.$$

rPS1 and rPS2 are the photosynthesis rate on day 1 and day 2, respectively; gFW2 is the fresh weight after gas exchange measurement; gFW1 is the objective term. To obtain µmolC_6_, gFW1 was converted to gDW (dry weight) with the factor 0.108 ± 0.0052 which was calculated from the ratio of dry and fresh weight of 81 plants. Based on elementary analysis from three replicates, 1 gDW contains 0.45 ± 0.024 g carbon. This was used as the only parameter implemented to the model to constrain shoot structural carbon formation after 24 h. Note that root biomass formation is not included in the model. The carbon devoted to root metabolism is contained in the export term of the model and not further resolved as maintenance and growth of the root.

### Dynamic modeling

To model the central carbohydrate metabolism in Arabidopsis leaves the following set of ordinary differential equations (ODE) was set up (ODEs of model 08, final).

$${\mathrm d}/{\mathrm d}t{\mathrm {HP}} = 1/6 \ast r1 - r3 - r4 - r5 + r7 + r8 - r15 - r14 - r11,$$

$${\mathrm d}/{\mathrm d}t{\mathrm {Starch}} = r3 + r4,$$

$${\mathrm d}/{\mathrm d}t{\mathrm {BM}} = r10 + r11,$$

$${\mathrm d}/{\mathrm d}t{\mathrm {exp}} = 2 \ast r13 + r12,$$

$${\mathrm d}/{\mathrm d}t{\mathrm {Suc}} = 1/2 \ast r5 - r6 - r13,$$

$${\mathrm d}/{\mathrm d}t{\mathrm {Glc}} = r6 - r7,$$

$${\mathrm d}/{\mathrm d}t{\mathrm {Frc}} = r6 - r8,$$

$${\mathrm d}/{\mathrm d}t{\mathrm {Aa}} = r9 - r10 - r12,$$

$${\mathrm d}/{\mathrm d}t{\mathrm {Cit}} = r14 - r9 - r16 + r17,$$

$${\mathrm d}/{\mathrm d}t{\mathrm {MF}} = r15 + 1/6 \ast r2 + r16 - r17.$$

A detailed list of rate equation descriptions and corresponding citations can be found in Table 4 and a full model description can be found as supplementary text file (Text_S1, “Final model”). According to Rohwer and Botha^49^ as well as Nägele et al.^24^ terms for enzyme inhibition for the modeled Michaelis–Menten kinetics were applied. Hexokinase activity was modeled with competitive inhibition by the hexose (fructose for glucokinase and glucose for fructokinase). Invertase was modeled with fructose as a competitive inhibitor and glucose as noncompetitive inhibitor.^47^ *K*_M_ values were modeled as a constant parameter during the 24 h cycle, whereas the maximal reaction rates (*V*_m_) were adjustable within the measured borders (*V*_m_ ± SD, see Fig. S10). To adjust *K*_M_ for competitive inhibition we implemented inhibition terms that modify the *K*_M_ value by taking the actual substrate concentration into account (see Table 4 for more details). Reaction rates were doubled according to the RGT rule for the high light models as the temperature was almost 10 °C higher under this condition. Interconversions without defined enzyme kinetics were modeled as mass balance equations (*r*9–*r*17). Stoichiometric factors were applied when metabolite interconversions included a change in the number of C atoms of substrates and products: all metabolites were expressed as C_6_-body, except for sucrose that is expressed as C_12_ and CO_2_, which is C_1_. Thus, the rates of photosynthesis (*r*1) and respiration (*r*2) are expressed as C1, while hexose phosphates are C_6_ metabolites. Therefore the rate for hexose phosphate formation was expressed as 1/6 *r*1; the same calculation was done for respiration. For HP to Suc and for Suc to Exp the stoichiometric factor is 2, as 1/2 Suc can be formed from one HP and two C_6_ export bodies can be formed from one Suc molecule. Rates for photosynthesis and respiration were calculated from measure data as smoothing spline over 24 h. Starch synthesis during the day and degradation during the night were calculated separately by the first derivative of the interpolation of the measured data giving a positive sign for starch synthesis and a negative sign for its degradation and zeros for synthesis during the night and degradation during the day. Detailed description of model parameters can be found in Table 4. Unknown parameters were identified by minimizing the cost function (sum of squared errors between simulated and measured data). This was performed using a particle-swarm pattern search method for bound constrained global optimization^50^ implemented in the software packages Systems Biology Toolbox2 and the SBPD Extension Package^51^ for the numerical software Matlab® (version R2014a)

**Table 4:** Description of parameters and constraints used in the models and their respective source whenever available

Name	Symbol	Constraints/range	Reference/note
SPS K_M_	K_M_5	0.1– 2	^25, 52, 53^^,^
			*Spinacia oleracea*
			*Pisum sativum*
Inv K_M_	K_M_6	7–13	^54^
			*Arabidopsis thaliana* Oy-0
Hxk K_M_ for GlcK activity	K_M_7	1.1–0.2	^55, 56^
		(0.01–2 for *gin2-1*)	*Helianthus annuus*, various
Hxk K_M_ for FrcK activity	K_M_8	0.1–3 (0.1–5 for *gin2-1*)	^55, 56^
			*Helianthus annuus*, various
Inv K_i_ for Frc inhibition	K_i_6a	0.001–10	^57^
			*Lolium temulentum*
Inv K_i_ for Glc inhibition	K_i_6b	0.001–10	Assumed and optimized
Hxk K_i_ for Frc inhibition	K_i_7	0.01–10	Assumed and optimized
Hxk K_i_ for Glc inhibition	K_i_8	0.001–10	Assumed and optimized
SPS maximal reaction rate	V_m_5	10–20 for both	Measured
Inv maximal reaction rate	V_m_6	60–100 for Ler	Measured
		70–120 for *gin2-1*	
GlcK maximal reaction rate	V_m_7	2–4 for Ler	Measured
		0–1 for *gin2-1*	
FrcK maximal reaction rate	V_m_8	5–10 for Ler	Measured
		2–5 for *gin2-1*	
Photosynthesis rate	*r*1		Measured and splined
respiration rate	*r*2	−28.6 for Ler N	Measured
		−37.5 for *gin2-1* N	
		−44.0 for Ler HL	
		−47.5 for *gin2-1* HL	
Rate of starch synthesis	*r*3	1–7.5 for N	Measured and splined
		5–26 for HL	
Rate of starch degradation	*r*4	−4 to −10 for N	Measured and splined
		−6 to −20 for HL	
SPS activity as MM	*r*5	(*V*_m_5∙HP)/(km5 + HP)	Measured and splined
Inv activity as MM	*r*6	(*V*_m_6∙Suc)/((km6∙(1 + Frc/Ki6a) + Suc)∙(1 + Glc/Ki6b))	Measured and splined^24,47,49^
GlcK activity as MM	*r*7	(V_m_7 ∙Glc)/(km7 ∙(1 + Frc/Ki7) + Glc)	Measured and splined^49^
FrcK activity as MM	*r*8	(V_m_8 ∙Frc)/(km8∙ (1 + Glc/Ki8) + Frc)	Measured and splined^49^
Aa synthesis from Cit as MBE	*r*9	r_ca ∙Cit	Assumed and optimized
		r_ca ∈ {0.01,100}	
SC formation from Aa as MBE	*r*10	a_ba∙Aa	Assumed and optimized
		a_ba ∈ {0,1}	
SC formation from HP as MBE	*r*11	ab_hp∙HP	Assumed and optimized
		ab_hp ∈ {0.0001,100}	
Exp rate from Aa as MBE	*r*12	aa_e∙Aa	Assumed and optimized
		aa_e ∈ {0.0001,10}	
Exp rate from Suc as MBE	*r*13	a_e∙Suc	Assumed and optimized
		a_e ∈ {0.0001,10}	
Cit formation from HP as MBE	*r*14	hp_c∙HP	Assumed and optimized
		hp_c ∈ {0.01,10}	
MF formation from HP as MBE	*r*15	hp_mf∙HP	Assumed and optimized
		hp_mf ∈ {0.01,10}	
MF formation from Cit as MBE	*r*16	cit_mf∙Cit	Assumed and optimized
		cit_mf ∈ {0.01,10}	
Cit formation from MF as MBE	*r*17	mf_cit∙MF	Assumed and optimized
		mf_cit ∈ {0.01,10}	

*SPS* sucrose-phosphate-synthase, *Inv* invertase, *Hxk* hexokinase, *GlcK* glucokinase, *FrcK* fructokinase, *Frc* fructose, *Glc* glucose, *HP* hexose phosphate, *Exp* export, *SC* structural carbon, *Suc* sucrose, *Cit* citrate, *MF* malate/fumarate, *Aa* amino acids, *MM* Michaelis–Menten kinetic, *MBE* mass balance equation. *K*_M_ are given in mM and *V*_m_ in µmol h^−1^ gFW^−1^

### Data analysis and statistics

Data evaluation, normalization, and visualization were performed in Microsoft Excel® (Microsoft Office version 2010, http://www.microsoft.com) and the numerical software Matlab (version R2014a). Analysis of variance with Tukey’s HSD (Honestly Significant Difference) test using a significance cutoff of *P* < 0.05 were performed with the R software (The R Project for Statistical Computing; http://www.r-project.org/).

## Supplementary information

Supplementary Figures

Supplementary Materials

## Data Availability

Raw data can be made available by the authors upon request.

## Supplementary information

**Supplementary information** accompanies the paper on the *npj Systems Biology and Applications* website (10.1038/s41540-018-0080-1).
