# Supplementary material for: Mathematical modeling of diurnal patterns of carbon allocation to shoot and root in Arabidopsis thaliana
Source: NPJ Syst Biol Appl. 2019 Jan 24;5:4. doi: 10.1038/s41540-018-0080-1 (PMC6346032; doi:10.1038/s41540-018-0080-1)
Supplement: Supplementary file 1 — Supplementary Figures [file 41540_2018_80_MOESM1_ESM.pdf]

Supporting Information

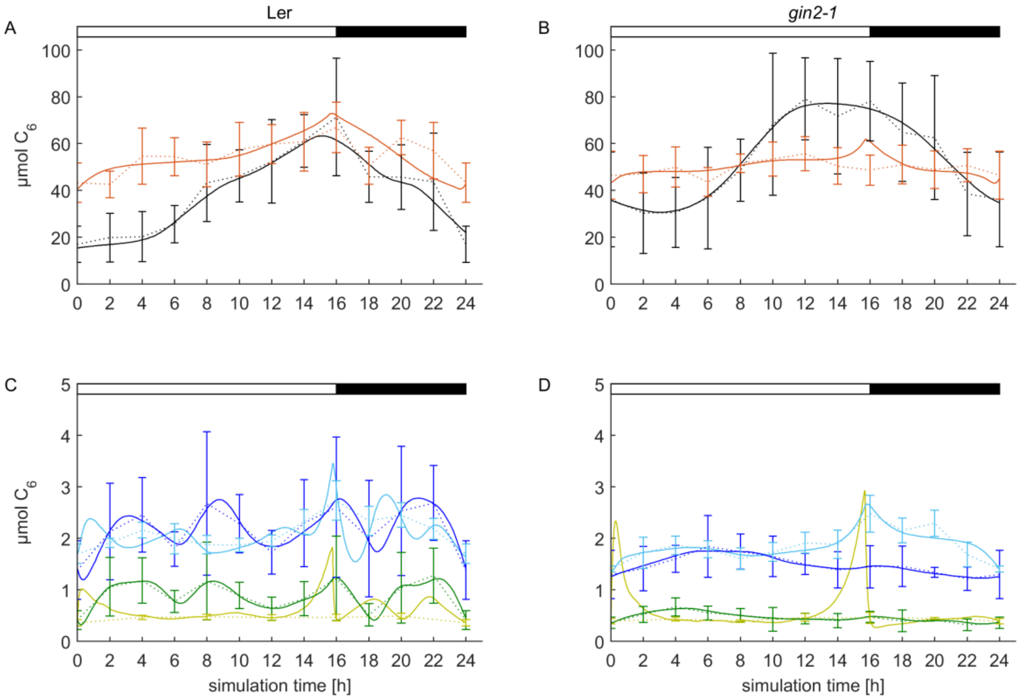

Figure S1: Simulated results of the basic model with one combined carbonic- and amino acid pool (Model_01). Dotted lines represent means of measured data ± SD (n=6), solid lines represent means of model simulations (n=5) for Ler (A, C) and *gin2-1* (B, D) under control conditions. Day and night are indicated with white and black bars on top.

[A,B] starch (black), carbonic- and amino acids pool (orange)

[C,D] glucose (blue), sucrose (turquois), fructose (green) and hexose-phosphates (olive)

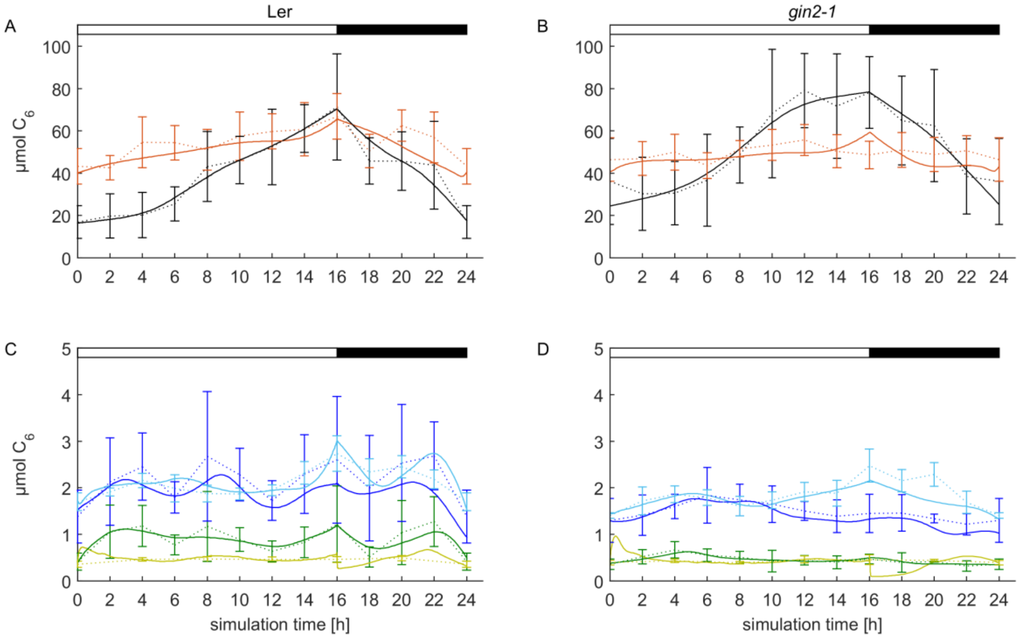

Figure S2: Simulated results of the basic model with one combined carbonic- and amino acid pool after separated splines for starch synthesis (day) and starch degradation (night) (Model_02). Dotted lines represent means of measured data ± SD (n=6), solid lines represent means of model simulations (n=5) for Ler (A, C) and *gin2-1* (B, D) under control conditions. Day and night are indicated with white and black bars on top.

[A,B] starch (black), carbonic- and amino acids pool (orange)

[C,D] glucose (blue), sucrose (turquois), fructose (green) and hexose-phosphates (olive)

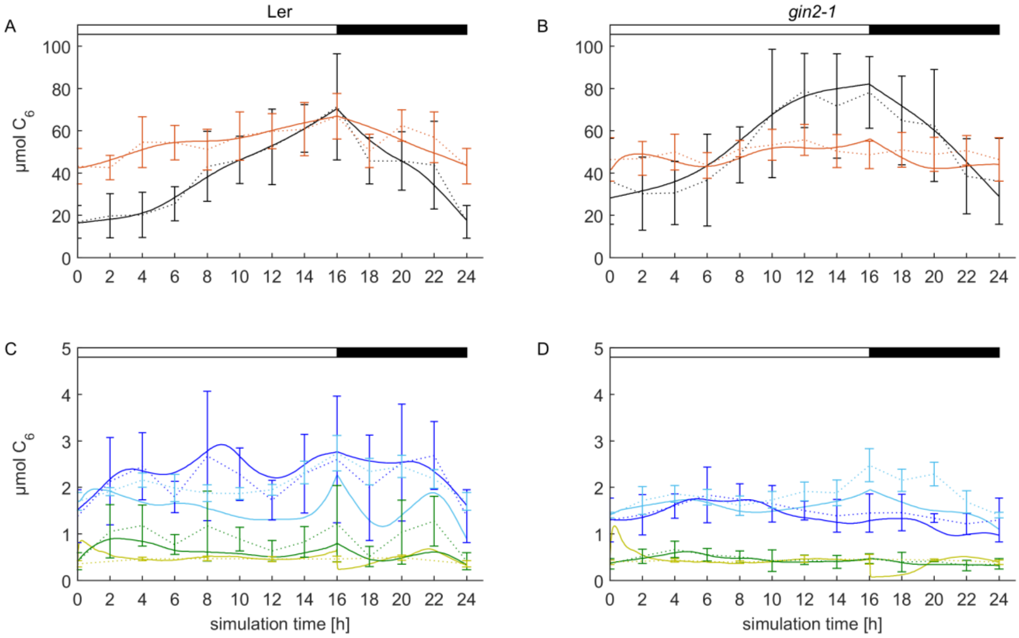

Figure S3: Simulated results of the basic model with one combined carbonic- and amino acid pool after separated splines for starch synthesis (day) and starch degradation (night) and a constant respiration rate during the day (Model_03). Dotted lines represent means of measured data ± SD (n=6), solid lines represent means of model simulations (n=5) for Ler (A, C) and *gin2-1* (B, D) under control conditions. Day and night are indicated with white and black bars on top.

[A,B] starch (black), carbonic- and amino acids pool (orange)

[C,D] glucose (blue), sucrose (turquois), fructose (green) and hexose-phosphates (olive)

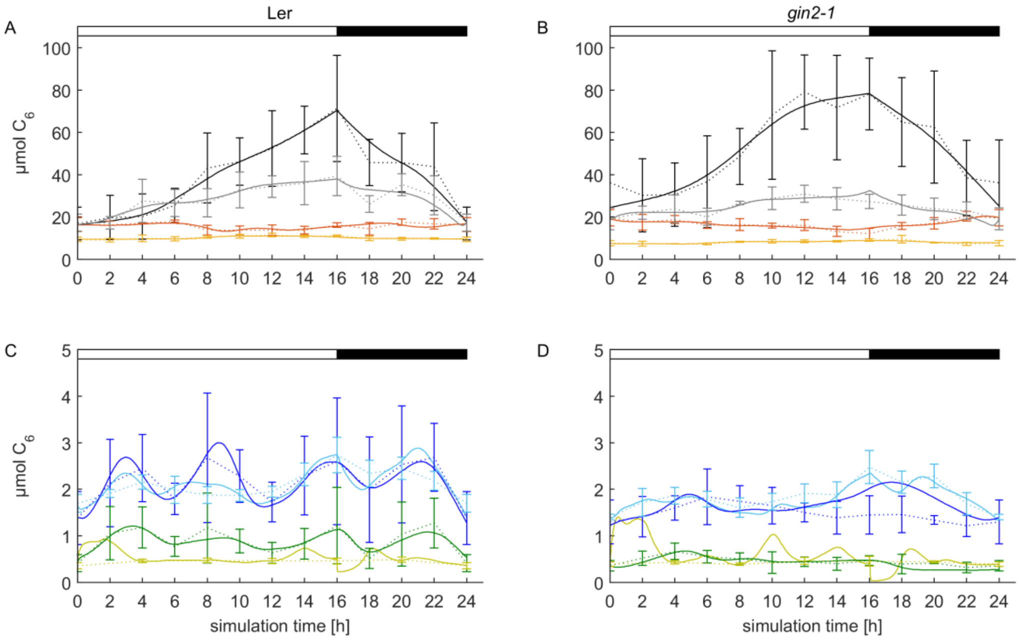

Figure S4: Simulated results after splitting the carbon pool into carbonic acids and amino acids (Model_04). Dotted lines represent means of measured data ± SD (n=6), solid lines represent means of model simulations (n=5) for Ler (A, C) and *gin2-1* (B, D) under control conditions. Day and night are indicated with white and black bars on top.

[A,B] starch (black), malate/fumarate pool (grey), citrate (orange) and amino acids (yellow)

[C,D] glucose (blue), sucrose (turquois), fructose (green) and hexose-phosphates (olive)

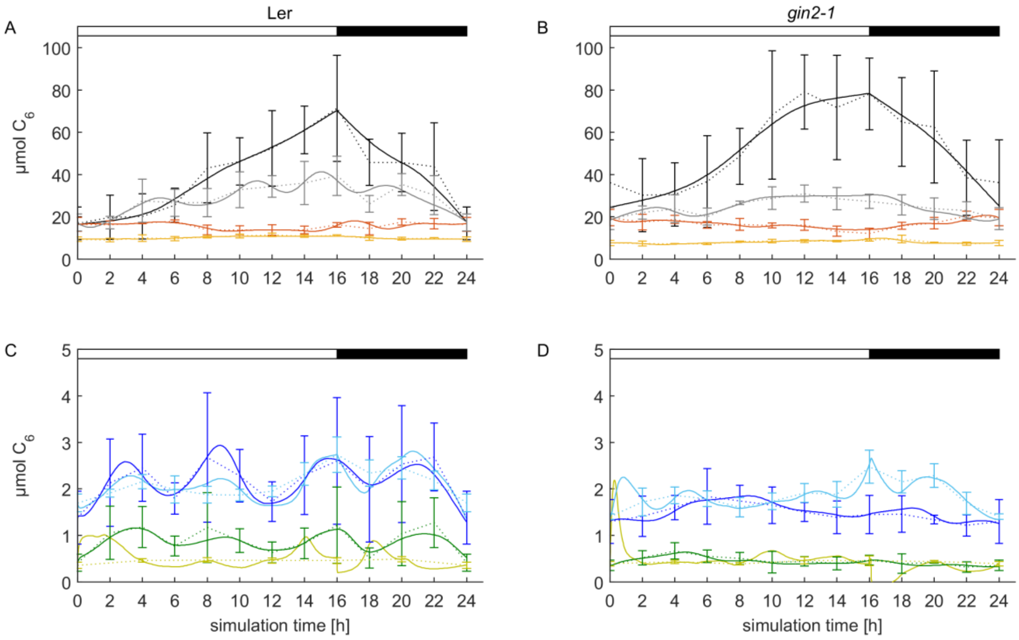

Figure S5: Simulated results after introduction of an exchange between citrate and the malate/fumarate pool (Model_05). Dotted lines represent means of measured data ± SD (n=6), solid lines represent means of model simulations (n=5) for Ler (A, C) and *gin2-1* (B, D) under control conditions. Day and night are indicated with white and black bars on top.

[A,B] starch (black), malate/fumarate pool (grey), citrate (orange) and amino acids (yellow)

[C,D] glucose (blue), sucrose (turquois), fructose (green) and hexose-phosphates (olive)

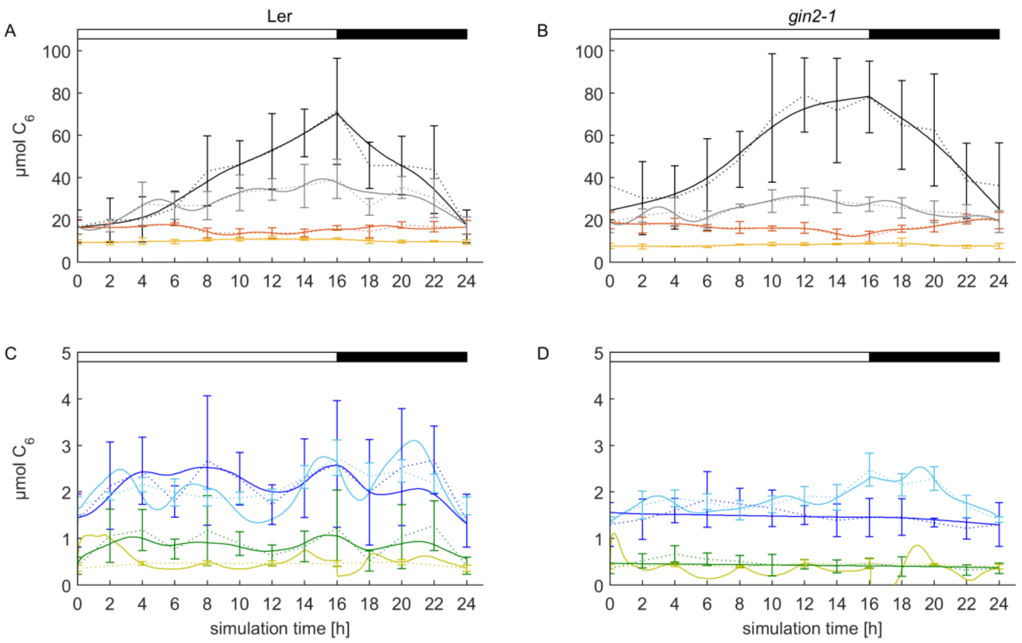

Figure S6: Simulated results after modeling the invertase, saccharose-phosphate-synthase and the fructose- and glucokinase activity as spline (Model_06). Dotted lines represent means of measured data ± SD (n=6), solid lines represent means of model simulations (n=5) for Ler (A, C) and *gin2-1* (B, D) under control conditions. Day and night are indicated with white and black bars on top.

[A,B] starch (black), malate/fumarate pool (grey), citrate (orange) and amino acids (yellow)

[C,D] glucose (blue), sucrose (turquois), fructose (green) and hexose-phosphates (olive)

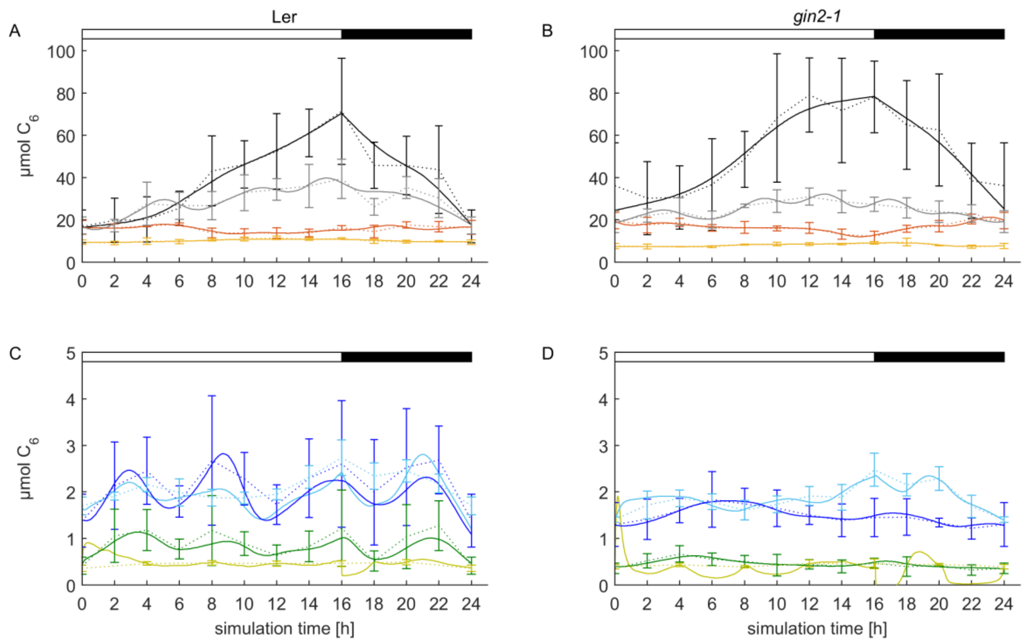

Figure S7: Simulated results after modeling the invertase and saccharose-phosphate-synthase activity as spline (Model_07). Dotted lines represent means of measured data ± SD (n=6), solid lines represent means of model simulations (n=5) for Ler (A, C) and *gin2-1* (B, D) under control conditions. Day and night are indicated with white and black bars on top.

[A,B] starch (black), malate/fumarate pool (grey), citrate (orange) and amino acids (yellow)

[C,D] glucose (blue), sucrose (turquois), fructose (green) and hexose-phosphates (olive)

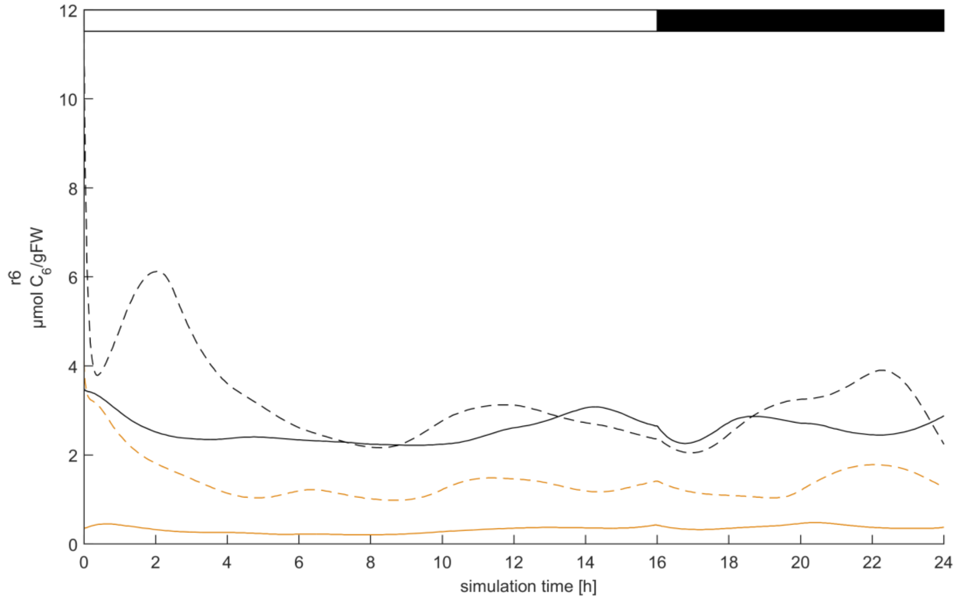

Figure S8: Simulated rates of the final model 08 for the invertase activity (r6) in Ler (black) and *gin2-1* (orange) under control (solid lines) and high light (dashed lines) conditions. Day and night are indicated with white and black bars on top.

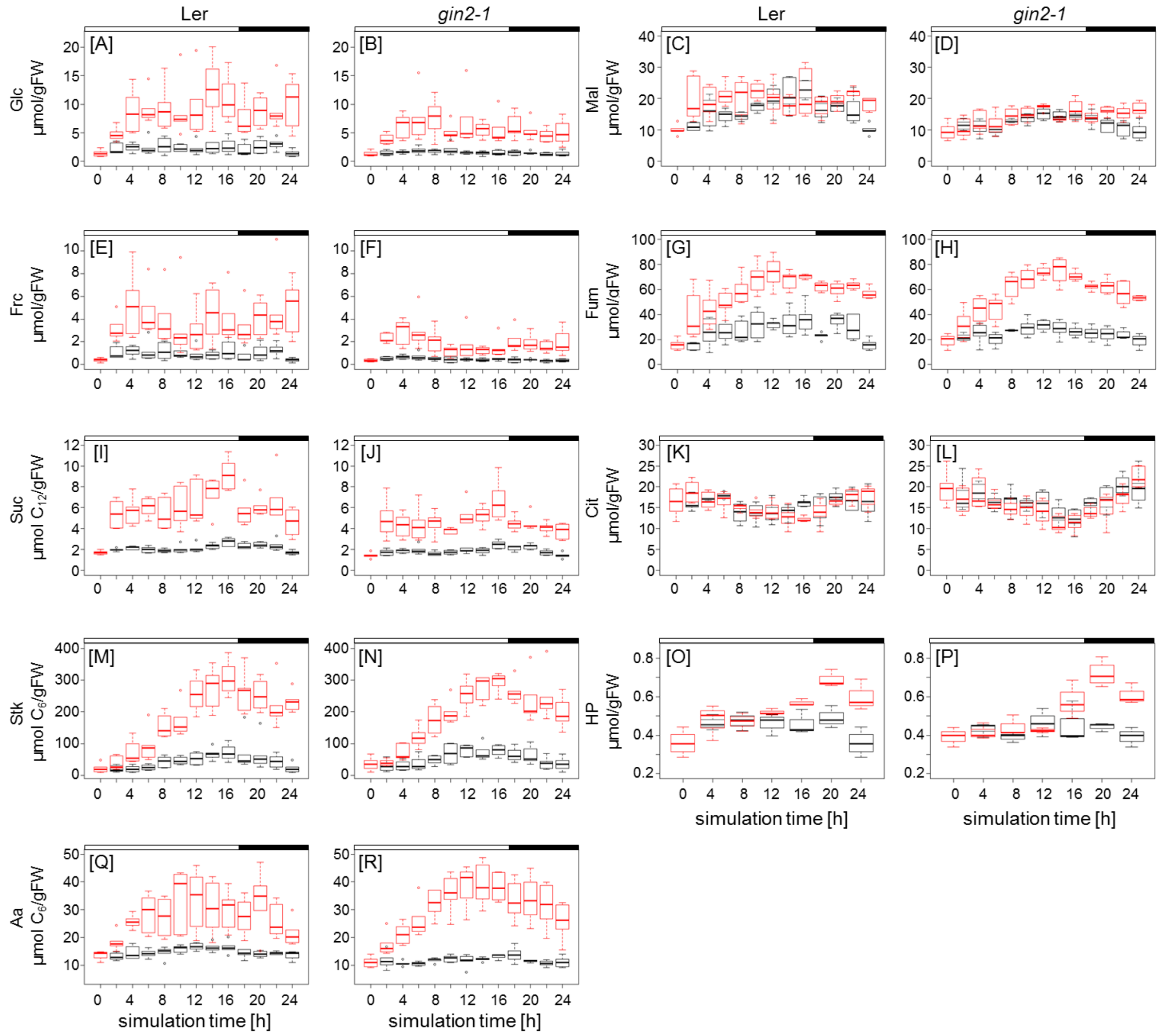

Figure S9: Boxplots of measured metabolite concentrations (n=6) for Ler and *gin2-1* under control (black) and high light (red) conditions. Depicted is Ler in the first and third column and *gin2-1* in the second and fourth column. Starch and amino acids are measured in C_6, and sucrose in C_{12}. Day and night are indicated with white and black bars on top. Glc: glucose, Frc: fructose, Suc: sucrose, Mal: malate, Fum: fumarate, Cit: citrate, Stk: starch, HP: hexose-phosphate, Aa: amino acids.

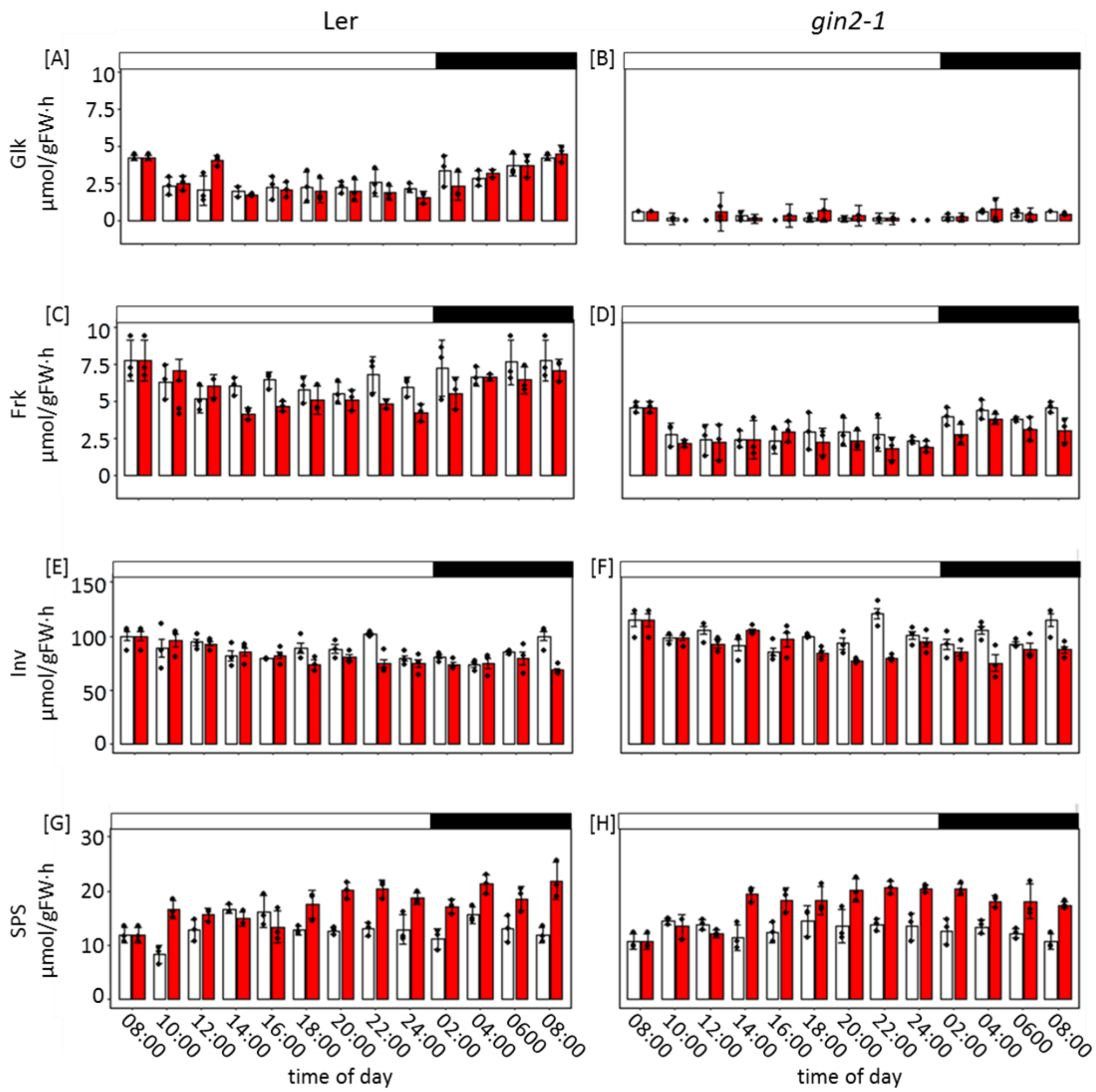

Figure S10: Bar graphs of measured maximal reaction rates (n=3 ± SD) for Ler and *gin2-1* under control (white) and high light (red) conditions. Individual data points are included as black dots over the respective bar graph, due to similar values of measured data points less than three dots can be visible. Day and night are indicated with white and black bars on top. Glk: glucokinase, Frk: fructokinase, Inv: invertase (acidic and neutral), SPS: sucrose-phosphate-synthase.
