# Supplementary material for: Mathematical modeling of diurnal patterns of carbon allocation to shoot and root in Arabidopsis thaliana
Source: NPJ Syst Biol Appl. 2019 Jan 24;5:4. doi: 10.1038/s41540-018-0080-1 (PMC6346032; doi:10.1038/s41540-018-0080-1)
Supplement: Supplementary file 2 — Supplementary Materials [file 41540_2018_80_MOESM2_ESM.pdf]

Supplementary material S1 -Model development-

Model characteristics

Initially the measured data were evaluated with an already existing, basic model published by Nägele *et al.*[1] to confirm consistency of our data. Some problems were encountered, like the overshoot of the HP pool at the day/night transition. Within the next steps we resolved this problem by introducing an explicit toggle between starch synthesis during the day and degradation during the night. This was achieved by separately splining synthesis and degradation. Additionally, the respiration rate was kept constant throughout the whole 24 h cycle and CO_2 uptake was adjusted accordingly, thus to reach the measured NPS. This was motivated by the assumption that mitochondrial respiration is necessary to provide reducing equivalents for nitrogen fixation during the day[2,3]. Further progress was achieved by dividing combined metabolite pools into individual components, which enabled a more precise simulation of their dynamics. For example, the combined CaAa pool was divided into an Aa, Cit and Mal/Fum pool. Separation of Cit from Mal/Fum was an important step, because Mal/Fum show the typical behavior of storage metabolites, while Cit is described as C-source for Aa synthesis[4,5] and has thus deviating diurnal dynamics. It was also necessary to implement cycling between Cit and Mal/Fum according to the TCA cycle reactions, which is likewise accepted in several other models[5–8]. With increasing model complexity simulation times rose significantly and almost doubled from 3 - 4 hours up to 6 - 7 hours. In the models 1 to 5, the maximal reaction rate of enzymes was optimized for each time step within the borders defined by measured values (mean ± standard deviation, see Fig. S9). In order to reduce computing time, the maximal reaction rates of the enzymes were codified by a smoothing spline through the measured values and the splined values for discrete time points were used for modeling. This nearly halved the computing time. With splines for all maximal reaction rates of sucrose cycle enzymes, hexose dynamics were wiped out, especially for the *gin2-1* mutant. However, as soon as parameter estimation was allowed for the hexokinases, hexose dynamics were recovered. We thus decided to keep the parameter estimation for hexokinase, because these parameters are also the main difference between Ler and the *gin2-1* mutant. To finally estimate the allocation of excess carbon to sink export or source structural carbon formation, we included the calculated structural carbon gain after 24 h as an endpoint that had to be reached by the model and introduced a mass balance equation from HP and Aa to structural carbon and export. No further restrictions were made for modeling structural carbon formation and C-export, and therefore the course of the curve is solely the result of parameter optimization.

Final model

```
********** MODEL NAME
final_model_Ler_N

********** MODEL NOTES
unit: C6/gFW (Suc in C12, r1 and r2 in C1)

Full model description of the final model for Ler under control conditions
with optimized parameters.

This model can be run with the software packages Systems Biology Toolbox2
and the SBPD Extension Package for the numerical software Matlab®



********** MODEL STATES
d/dt(HP) = (1/6)*r1-r3-r4-r5+r7+r8-r15-r14-r11
d/dt(Starch) = r3+r4
d/dt(SC) = r10+r11
d/dt(Exp) = 2*r13+r12
d/dt(Suc) = (1/2*r5)-r6-r13
d/dt(Glc) = r6-r7
d/dt(Frc) = r6-r8
d/dt(Aa) = r9-r10-r12
d/dt(Cit) = r14-r9-r16+r17
d/dt(MF) = r15+(1/6)*r2+r16-r17



HP(0) =     0.359051
Starch(0)   =     16.412442
SC(0) =     1.389453
Exp(0)      =     0.997836
Glc(0)      =     1.352510
Frc(0)      =     0.412193
Suc(0)      =     1.670430
Aa(0) =     9.414735
Cit(0)      =     16.454939
MF(0) =     17.193958



********** MODEL PARAMETERS
a_e_1 =     0.0001
a_e_2 =     0.0001
a_e_3 =     0.368165425
a_e_4 =     0.456169235
a_e_5 =     0.55426045
a_e_6 =     0.592706043
a_e_7 =     0.0001
a_e_8 =     0.0001
a_e_9 =     0.0001
a_e_10      =     0.010641201
a_e_11      =     0.096178904
a_e_12      =     0.001
a_ba_1      =     0.01059224
a_ba_2      =     0.077026168
a_ba_3      =     0.392787105
a_ba_4      =     0.086143433
a_ba_5      =     0.030598025
```

a_ba_6 = 0.357565053
a_ba_7 = 0.094224894
a_ba_8 = 0.041214952
a_ba_9 = 0.11940543
a_ba_10 = 0.01
a_ba_11 = 0.001073722
a_ba_12 = 0.005459969
ab_hp_1 = 3.422898282
ab_hp_2 = 13.38299382
ab_hp_3 = 0.01
ab_hp_4 = 30.92012188
ab_hp_5 = 30.12948095
ab_hp_6 = 0.259551389
ab_hp_7 = 2.122535075
ab_hp_8 = 5.842423752
ab_hp_9 = 2.545853377
ab_hp_10 = 0.01
ab_hp_11 = 0.0001
ab_hp_12 = 0.912227824
aa_e_1 = 2.265679057
aa_e_2 = 0.260858434
aa_e_3 = 1.245502732
aa_e_4 = 0.158306785
aa_e_5 = 0.036514634
aa_e_6 = 0.764893662
aa_e_7 = 1.575983734
aa_e_8 = 0.806146148
aa_e_9 = 1.272239826
aa_e_10 = 0.025198784
aa_e_11 = 0.031902487
aa_e_12 = 0.273800589
km5 = 0.53368893
km6 = 13
km7 = 0.2
km8 = 0.1
ki6a = 10
ki6b = 0.646904378
ki7 = 9.900301865
ki8 = 0.175508718
vm7_1 = 4.173216913
vm7_2 = 2.142096312
vm7_3 = 3.045
vm7_4 = 2.356
vm7_5 = 2.084110868
vm7_6 = 3.246630687
vm7_7 = 2.52251306
vm7_8 = 3.371498497
vm7_9 = 2.459
vm7_10 = 3.46867234
vm7_11 = 2.456359095
vm7_12 = 2.97
vm8_1 = 9.192
vm8_2 = 5.144552701
vm8_3 = 5.534053046
vm8_4 = 6.088731182
vm8_5 = 5.893
vm8_6 = 6.300541966
vm8_7 = 6.303
vm8_8 = 7.911599487
vm8_9 = 5.548481731
vm8_10 = 9.138
vm8_11 = 6.036
vm8_12 = 6.099146267

hp_c_1	=	26.06928259
hp_c_2	=	0.538440672
hp_c_3	=	14.88881842
hp_c_4	=	2.519565316
hp_c_5	=	0.01
hp_c_6	=	12.24281651
hp_c_7	=	0.720118704
hp_c_8	=	6.261434777
hp_c_9	=	37.10283186
hp_c_10	=	4.068241267
hp_c_11	=	0.201772351
hp_c_12	=	14.81795569
hp_mf_1	=	0.01
hp_mf_2	=	13.02060902
hp_mf_3	=	36.02683262
hp_mf_4	=	17.14069134
hp_mf_5	=	7.017216768
hp_mf_6	=	47.43659624
hp_mf_7	=	42.79531375
hp_mf_8	=	30.10341102
hp_mf_9	=	3.538228619
hp_mf_10	=	10.42149161
hp_mf_11	=	3.430490625
hp_mf_12	=	25.19267534
r_ca_1	=	1.222287495
r_ca_2	=	0.133359091
r_ca_3	=	1.051923906
r_ca_4	=	0.091796378
r_ca_5	=	0.133616115
r_ca_6	=	0.878102865
r_ca_7	=	1.341995227
r_ca_8	=	0.683583622
r_ca_9	=	0.950535581
r_ca_10	=	0.01
r_ca_11	=	0.01
r_ca_12	=	0.210295005
cit_mf_1	=	0.01
cit_mf_2	=	0.175325042
cit_mf_3	=	0.01
cit_mf_4	=	0.01
cit_mf_5	=	0.054656412
cit_mf_6	=	0.117162819
cit_mf_7	=	0.557987136
cit_mf_8	=	0.01
cit_mf_9	=	0.01
cit_mf_10	=	0.01
cit_mf_11	=	0.023929186
cit_mf_12	=	0.01
mf_cit_1	=	0.538180735
mf_cit_2	=	0.020738016
mf_cit_3	=	0.497971674
mf_cit_4	=	0.01
mf_cit_5	=	0.01
mf_cit_6	=	0.327031054
mf_cit_7	=	0.746527562
mf_cit_8	=	0.165458371
mf_cit_9	=	0.01
mf_cit_10	=	0.01
mf_cit_11	=	0.01
mf_cit_12	=	0.01

```
********** MODEL VARIABLES
r1=interpcsSB([0,0.0333564975677554,0.0667129951355108,0.100069492703266,0.
133425990271022,0.166782487838777,0.200138985406532,0.233495482974288,0.266
851980542043,0.300208478109798,0.333564975677554,0.366921473245309,0.400277
970813065,0.433634468380820,0.466990965948575,0.500347463516331,0.533703961
084086,0.567060458651842,0.600416956219597,0.633773453787352,0.667129951355
108,0.700486448922863,0.733842946490618,0.767199444058374,0.800555941626129
,0.833912439193885,0.867268936761640,0.900625434329396,0.933981931897151,0.
967338429464906,1.00069492703266,1.03405142460042,1.06740792216817,1.100764
41973593,1.13412091730368,1.16747741487144,1.20083391243919,1.2341904100069
5,1.26754690757470,1.30090340514246,1.33425990271022,1.36761640027797,1.400
97289784573,1.43432939541348,1.46768589298124,1.50104239054899,1.5343988881
1675,1.56775538568450,1.60111188325226,1.63446838082001,1.66782487838777,1.
95135510771369,2.23488533703961,2.51841556636553,2.80194579569145,3.0854760
2501737,3.36900625434329,3.65253648366922,3.93606671299514,4.21959694232106
,4.50312717164698,4.78665740097290,5.07018763029882,5.35371785962474,5.6372
4808895066,5.92077831827658,6.20430854760250,6.48783877692842,6.77136900625
434,7.05489923558026,7.33842946490619,7.62195969423211,7.90548992355803,8.1
8902015288395,8.47255038220987,8.75608061153579,9.03961084086171,9.32314107
018763,9.60667129951355,9.89020152883947,10.1737317581654,10.4572619874913,
10.7407922168172,11.0243224461432,11.3078526754691,11.5913829047950,11.8749
131341209,12.1584433634468,12.4419735927728,12.7255038220987,13.00903405142
46,13.2925642807505,13.5760945100764,13.8596247394024,14.1431549687283,14.4
266851980542,14.7102154273801,14.9770674079222,15.0104239054899,15.04378040
30577,15.0771369006254,15.1104933981932,15.1438498957609,15.1772063933287,1
5.2105628908965,15.2439193884642,15.2772758860320,15.3106323835997,15.34398
88811675,15.3773453787352,15.4107018763030,15.4440583738707,15.477414871438
5,15.5107713690063,15.5441278665740,15.5774843641418,15.6108408617095,15.64
41973592773,15.6775538568450,15.7109103544128,15.7442668519805,15.777623349
5483,15.8109798471161,15.8443363446838,15.8776928422516,15.9110493398193,15
.9444058373871,15.9777623349548,16.0111188325226,16.0444753300903,16.077831
8276581,16.1111883252259,16.1445448227936,16.1779013203614,16.2112578179291
,16.2446143154969,16.2779708130646,16.3113273106324,16.3446838082001,16.378
0403057679,16.4113968033357,16.4447533009034,16.4781097984712,16.5114662960
389,16.5448227936067,16.5781792911744,16.6115357887422,16.6448922863099,16.
6782487838777,16.7116052814454,16.7449617790132,16.7783182765810,16.8116747
741487,16.8450312717165,16.8783877692842,16.9117442668520,16.9451007644197,
16.9784572619875,17.0118137595552,17.0451702571230,17.0785267546908,17.1118
832522585,17.3954134815844,17.6789437109104,17.9624739402363,18.24600416956
22,18.5295343988881,18.8130646282140,19.0965948575400,19.3801250868659,19.6
636553161918,19.9471855455177,20.2307157748436,20.5142460041696,20.79777623
34955,21.0813064628214,21.3648366921473,21.6483669214732,21.9318971507992,2
2.2154273801251,22.4989576094510,22.7824878387769,23.0660180681029,23.34954
82974288,23.6330785267547,23.9166087560806,24],[111.367417858534,143.878946
887327,150.643631242738,156.344769608933,160.974910133446,164.580849768140,
167.254147833396,169.113322937934,170.292618834923,170.938180434603,171.199
395526190,171.188452008203,170.981640103880,170.645689102258,170.2348839108
92,169.778119630879,169.286016247949,168.768783843136,168.235950061550,167.
686028387512,167.114143186502,166.514676999806,165.893195349547,165.2525787
87163,164.589140961090,163.903281974931,163.202436938557,162.494513907099,1
61.780627401408,161.060028601667,160.332182154999,159.609314939751,158.9186
03427143,158.300488747160,157.789078748117,157.398930595745,157.14892362279
9,157.057909107777,157.135607677471,157.384713774502,157.796303376965,158.3
29157278455,158.916605767201,159.492082196652,160.002780922132,160.42002170
0598,160.719001780303,160.871260063806,160.856581203818,160.662790571770,16
0.283655705746,153.598689240740,149.915094635906,150.237668593655,151.79718
6393416,152.201348454273,151.963097894833,152.611885882964,154.703552768835
,153.043721094039,153.927537648377,152.977878992052,152.588519901115,154.24
7572004415,155.005526500657,154.773653518849,154.824085778118,154.592332437
021,155.711552709027,155.516418258875,156.181787082984,154.061418802255,153
```

```
.847577633418,159.052012272145,157.898948316234,157.714570941695,157.025389
841975,158.195611831605,158.074822006698,159.893949238908,157.983602258515,
157.696433749005,158.763333646875,158.494970546250,158.952119069604,159.350
277858103,158.945151682779,159.327190399950,160.356534142332,159.8797336822
64,160.235574243593,160.346402198638,159.541721701487,161.316405047114,162.
023448186446,161.427094414994,162.567705805762,163.796473200722,163.7607272
53658,163.702969477990,163.646661340731,163.619017032515,163.648084959556,1
63.762379352745,163.989246592144,164.344311814130,164.832892721155,165.4506
71561870,166.191234386125,167.020174288690,167.894632689331,168.75096016674
9,169.514511939604,170.110501227575,170.464141250340,170.500836108437,170.1
52203073913,169.514511939604,170.110501227575,170.464141250340,170.50083610
8437,170.152203073913,169.514511939604,170.110501227575,170.464141250340,17
0.500836108437,170.152203073913,168.144914982732,0,0,0,0,0,0,0,0,0,0,0,0,0,
0,0,0,0,0,0,0,0,0,0,0,0,0,0,0,0,0,0,0,0,0,0,0,0,0,0,0,0,0,0,0,0,0,0,0,0,0,0
,0,0,0,0,0,0,0,0],time)

r3 =
interpcsSB([0,2,4,6,8,10,12,14,16,16.05,18,20,22,24],[0.850,0.954,2.286,4.7
84,4.780,3.302,3.589,4.468,4.856,0,0,0,0,0],time)

r4 =
interpcsSB([0,2,4,6,8,10,12,14,15.95,16.01,18,20,22,24],[0,0,0,0,0,0,0,0,0,
-7.983,-6.3580,-4.0873,-7.592,-8.743],time)

vm5 =
interpcsSB([0,2,4,6,8,10,12,14,16,18,20,22,24],[11.551,9.012,12.514,16.373,
16.007,13.162,12.600,12.950,12.540,12.105,14.697,13.318,11.551],time)

vm6 =
interpcsSB([0,2,4,6,8,10,12,14,16,18,20,22,24],[98.627,92.221,90.239,83.362
,81.121,86.490,91.838,95.085,84.630,78.105,76.213,83.345,98.627],time)

vm7 =
interpcsSB([0,2,4,6,8,10,12,14,16,18,20,22,24],[vm7_1,vm7_2,vm7_3,vm7_4,vm7
_5,vm7_6,vm7_7,vm7_8,vm7_9,vm7_10,vm7_11,vm7_12,vm7_1],time)

vm8 =
interpcsSB([0,2,4,6,8,10,12,14,16,18,20,22,24],[vm8_1,vm8_2,vm8_3,vm8_4,vm8
_5,vm8_6,vm8_7,vm8_8,vm8_9,vm8_10,vm8_11,vm8_12,vm8_1],time)

a_e =
interpcsSB([0,2,4,6,8,10,12,14,16,18,20,22,24],[a_e_1,a_e_2,a_e_3,a_e_4,a_e
_5,a_e_6,a_e_7,a_e_8,a_e_9,a_e_10,a_e_11,a_e_12,a_e_1],time)

aa_e =
interpcsSB([0,2,4,6,8,10,12,14,16,18,20,22,24],[aa_e_1,aa_e_2,aa_e_3,aa_e_4
,aa_e_5,aa_e_6,aa_e_7,aa_e_8,aa_e_9,aa_e_10,aa_e_11,aa_e_12,aa_e_1],time)

a_ba =
interpcsSB([0,2,4,6,8,10,12,14,16,18,20,22,24],[a_ba_1,a_ba_2,a_ba_3,a_ba_4
,a_ba_5,a_ba_6,a_ba_7,a_ba_8,a_ba_9,a_ba_10,a_ba_11,a_ba_12,a_ba_1],time)

ab_hp =
interpcsSB([0,2,4,6,8,10,12,14,16,18,20,22,24],[ab_hp_1,ab_hp_2,ab_hp_3,ab_
hp_4,ab_hp_5,ab_hp_6,ab_hp_7,ab_hp_8,ab_hp_9,ab_hp_10,ab_hp_11,ab_hp_12,ab_
hp_1],time)

hp_c =
interpcsSB([0,2,4,6,8,10,12,14,16,18,20,22,24],[hp_c_1,hp_c_2,hp_c_3,hp_c_4
,hp_c_5,hp_c_6,hp_c_7,hp_c_8,hp_c_9,hp_c_10,hp_c_11,hp_c_12,hp_c_1],time)

hp_mf =
interpcsSB([0,2,4,6,8,10,12,14,16,18,20,22,24],[hp_mf_1,hp_mf_2,hp_mf_3,hp_
```

```
mf_4,hp_mf_5,hp_mf_6,hp_mf_7,hp_mf_8,hp_mf_9,hp_mf_10,hp_mf_11,hp_mf_12,hp_
mf_1],time)

r_ca =
interpcsSB([0,2,4,6,8,10,12,14,16,18,20,22,24],[r_ca_1,r_ca_2,r_ca_3,r_ca_4
,r_ca_5,r_ca_6,r_ca_7,r_ca_8,r_ca_9,r_ca_10,r_ca_11,r_ca_12,r_ca_1],time)

cit_mf =
interpcsSB([0,2,4,6,8,10,12,14,16,18,20,22,24],[cit_mf_1,cit_mf_2,cit_mf_3,
cit_mf_4,cit_mf_5,cit_mf_6,cit_mf_7,cit_mf_8,cit_mf_9,cit_mf_10,cit_mf_11,c
it_mf_12,cit_mf_1],time)

mf_cit =
interpcsSB([0,2,4,6,8,10,12,14,16,18,20,22,24],[mf_cit_1,mf_cit_2,mf_cit_3,
mf_cit_4,mf_cit_5,mf_cit_6,mf_cit_7,mf_cit_8,mf_cit_9,mf_cit_10,mf_cit_11,m
f_cit_12,mf_cit_1],time)



********** MODEL REACTIONS
r5 = (vm5*HP)/(km5+HP)
r6 = (vm6*Suc)/((km6*(1+Frc/ki6a)+Suc)*(1+Glc/ki6b))
r7 = (vm7*Glc)/(km7*(1+Frc/ki7)+Glc)
r8 = (vm8*Frc)/(km8*(1+Glc/ki8)+Frc)


r10 = a_ba*Aa
r11 = ab_hp*HP

r12 = aa_e*Aa
r13 = a_e*Suc

r2 = -28.5955
r17 = mf_cit*MF
r16 = cit_mf*Cit

r9 = r_ca*Cit
r14 = hp_c*HP
r15 = hp_mf*HP




********** MODEL FUNCTIONS


********** MODEL EVENTS


********** MODEL MATLAB FUNCTIONS
```

References

1. Nägele, T. *et al.* Mathematical modeling of the central carbohydrate metabolism in Arabidopsis reveals a substantial regulatory influence of vacuolar invertase on whole plant carbon metabolism. *Plant physiology* **153,** 260–272 (2010).

2. Avelange, M.-H., Thiéry, J. M., Sarrey, F., Gans, P. & Rébeillé, F. Mass-spectrometric determination of O2 and CO2 gas exchange in illuminated higher-plant cells. *Planta* **183,** 150–157 (1991).

3. Gerbaud, A. & Andre, M. Effect of CO2, O2, and Light on Photosynthesis and Photorespiration in Wheat. *Plant physiology* **66,** 1032–1036 (1980).

4. Cheung, C. M. Y., Poolman, M. G., Fell, D. A., Ratcliffe, R. G. & Sweetlove, L. J. A Diel Flux Balance Model Captures Interactions between Light and Dark Metabolism during Day-Night Cycles in C3 and Crassulacean Acid Metabolism Leaves. *Plant physiology* **165,** 917–929 (2014).

5. Hanning, I. & Heldt, H. W. On the Function of Mitochondrial Metabolism during Photosynthesis in Spinach (Spinacia oleracea L.) Leaves (Partitioning between Respiration and Export of Redox Equivalents and Precursors for Nitrate Assimilation Products). *Plant physiology* **103,** 1147–1154 (1993).

6. Steuer, R. *et al.* From structure to dynamics of metabolic pathways: application to the plant mitochondrial TCA cycle. *Bioinformatics* **23,** 1378–1385 (2007).

7. Igamberdiev, A. U. & Eprintsev, A. T. Organic Acids. The Pools of Fixed Carbon Involved in Redox Regulation and Energy Balance in Higher Plants. *Frontiers in plant science* **7,** 1042 (2016).

8. Nunes-Nesi, A., Sweetlove, L. J. & Fernie, A. R. Operation and function of the tricarboxylic acid cycle in the illuminated leaf. *Physiologia plantarum* **129,** 45–56 (2007).
